# Numerical investigation for hazardous gas cloud form and dissipation behaviour of hydrogen-blended natural gas in a confined space

**DOI:** 10.1098/rsos.241671

**Published:** 2025-02-19

**Authors:** Shuangqing Chen, Minghao Li, Hongli Dong, Lan Meng, Bing Guan, Shun Zhou, Shanlong Wang, Chaofan Niu

**Affiliations:** ^1^ School of Petroleum Engineering, Northeast Petroleum University, Daqing, People’s Republic of China; ^2^ Post-Doctoral Programme, Daqing Oilfield Co. Ltd, Daqing, People’s Republic of China; ^3^ Shandong Provincial Key Laboratory of Oil, Gas and New Energy Storage and Transportation Safety, China University of Petroleum (East China), Qingdao, People’s Republic of China; ^4^ Artificial Intelligence Energy Research Institute, Northeast Petroleum University, Daqing, People’s Republic of China; ^5^ Daqing Oilfield Design Institute Co. Ltd, Daqing, People’s Republic of China; ^6^ Key Laboratory of Continental Shale Hydrocarbon Accumulation and Efficient Development (Northeast Petroleum University), Ministry of Education, Daqing, People’s Republic of China

**Keywords:** hydrogen-blended natural gas, computational fluid dynamics simulation, gas leakage, hazardous gas cloud, hazardous gas ventilation

## Abstract

The safety of hydrogen-blended natural gas (HBNG) in a confined space is an issue, especially for ventilation processes. In this study, leakage and ventilation processes of low-pressure HBNG with different hydrogen-blended ratio (HBR) in a confined space are simulated and validated by experiment based on similarity criteria. For the leakage process, the leak direction and HBR do not significantly affect gas accumulation behaviour. The required time for a gas cloud to fill space decreases slightly with HBR rising and they generally show a linear relationship. For the ventilation process, the main influences on the leakage process are the total leakage mass and the ventilation conditions. The required time for hazardous gas cloud dissipation increases with total leakage mass and decreases with HBR. For different ventilation conditions, the ranking of required time to exhaust leaked gas is low > centre > high > mix. Through the analysis of pressure distribution, it is found time difference is produced by different airflow patterns. With the asymmetric layout, outside air rushes into the confined space from the high side and then flows out from the low side carrying the leaked HBNG. These findings inform the design of ventilation for HBNG utilization scenarios like restaurant facing the street.

## 1. Introduction

New energy source application will be an important part of future industrial systems [1]. Hydrogen energy is a zero-carbon emission energy source. It will be an important supplement to traditional fossil fuels in the future [2]. However, the storage and transportation of hydrogen are still a challenge [3]. At present, the main methods for delivering hydrogen energy include liquid tank, H_2_ trailer and H_2_ pipeline. These methods have high investment costs and low transportation efficiency [4]. In recent years, the transportation of hydrogen blended with natural gas through existing pipelines has been realized. With a reasonable hydrogen blending ratio (HBR), it achieves reduction in cost and the probability of pipeline failure [4,5]. Small-scale experimental projects have been conducted in many countries. With the gradual application of hydrogen-blended natural gas (HBNG), safety is a critical issue that needs to be discussed. H_2_ has a wider explosive limit range and lower ignition energy than CH_4_ [6,7]. Therefore, in order to reduce the risk of fire and explosion accidents, the leakage problem needs to be studied for the application of HBNG.

The HBNG leakage studies that have been conducted are mainly based on different scenarios. The conditions of leakage experiments are difficult to realize and also risky for researchers. Therefore, the numerical simulation method of computational fluid dynamics (CFD) is widely applied in HBNG leakage studies. Sun *et al*. simulated the HBNG leakage process in a semi-closed scenario and analysed the effects of wind speed and HBR on the diffusion range. Suggestions were developed for the installation of CH_4_ and H_2_ detectors [8]. Li *et al*. selected a full-closed scenario for leakage numerical simulation. The variation of flammable area thickness at different HBRs was mainly analysed [9]. Some other researchers conducted investigations of application scenarios with more complex geometry conditions. Lowesmith *et al*. analysed the leakage process of HBNG through full-scale experiments. This work obtained a mathematical model to describe the formation of a mixture layer in the upper part of a house [10]. Jia *et al*. simulated the HBNG leakage process inside the valve chamber of a natural gas pipeline. The variation characteristics of gas accumulation thickness were analysed under different conditions [11]. Su *et al*. simulated the process of HBNG leakage in a household kitchen. The variation of response time of gas alarms under different HBRs was mainly analysed [12].

Wang *et al*. and Han *et al*. simulated the leakage process of a HBNG pipeline in an urban underground tunnel. The variation of distribution range under natural and mechanical ventilation conditions was mainly analysed [13,14]. Based on the explosion limit division, the ventilation schemes to maintain safety were proposed. In addition, the difference in physical properties between CH_4_ and H_2_ may also have some influence on the distribution of leaked gas. Liu *et al*. conducted experiments and simulations on the flow of HBNG through a horizontal pipe. The phenomenon of CH_4_ and H_2_ stratification due to gas density difference was analysed [15]. Gas stratification was also observed in the static experiments conducted in confined containers [16,17].

**Table 1.** Nomenclature.

ρ	density	t	time
U	velocity vector	p	pressure
τ	stress tensor	$f_{b}$	body force
μ	dynamic viscosity	$\omega_{i}$	mass fraction of *i*
Ι	identity matrix	$D_{i}$	diffusion coefficient
$R_{i}$	mass source term	λ	thermal conductivity
$c_{p}$	specific heat capacity	${\overset{˙}{q}}_{v}$	unit thermal generation rate
k	turbulence energy	ω	specific dissipation rate
$\mu_{t}$	turbulence viscosity	$G_{k}$	generation terms of *k*
$G_{\omega }$	generation terms of *ω*	$G_{b}$	buoyancy term
$G_{\omega b}$	buoyancy term	$Y_{k}$	dissipation term of *k*
$U_{exit}$	gas velocity	d	leakage diameter
$\rho_{\infty }$	ambient air density	$\rho_{exit}$	leakage gas density
g	acceleration due to gravity	$Sc_{t}$	turbulence Schmidt number

Summarizing the above-mentioned studies, it can be concluded that leakage processes have been quite adequately studied for various scenarios. Most of them analyse the distribution characteristics of leaked gas. The characteristics are mainly on the thickness of flammable area, the leaked gas concentration and the range of leaked gas distribution. For the chain of gas leakage accident, a gas cloud explosion is the final accident with the greatest damage. The size of flammable gas clouds can significantly affect the consequences of an explosion [18]. There are fewer quantitative studies on the evolution of hazardous gas clouds. This deficiency needs to be analysed in more detail.

The ability of H_2_ leakage diffusion in an open space exceeds that of CH_4_. It makes HBNG rather less likely to accumulate when leaking in an open space. When the leak pressure is not high enough to trigger spontaneous hydrogen combustion, this can be considered a positive factor in favour of safety. However, many low-pressure scenarios concern confined spaces. Hydrogen diffusion capacity cannot be a positive factor. In some restaurants on the street, the kitchens are often divided arbitrarily by the owners. Their ventilation and fire safety cannot be maintained. These realities are difficulties when promoting HBNG for application, which is more dangerous than natural gas. When a leak occurs, the leaked gas will certainly accumulate into a hazardous gas cloud in the confined space until it is detected. Then the windows will be open to dissipate the hazardous gas cloud. For this reason, besides the leakage process, it is necessary to conduct research on the process of venting out the leaked gas. The ability to vent leaked gas fast in various scenarios will improve safety to some extent.

However, the venting process of leaked gas is difficult to monitor accurately. Experiments can be conducted to obtain discrete information on concentration changes at the monitoring site. But it does not quantify the evolution of the hazardous gas cloud over the entire space. It is difficult to conduct visualization studies of clouds formed by gas leakage experiments. Therefore, the CFD method can be used to study the accumulation and dissipation behaviour of leaked gas. Li *et al*. have conducted H_2_ leakage CFD simulation in a tunnel and analysed the gas cloud characteristics. This validates the feasibility of CFD simulations in this type of study [19].

In this study, low-pressure HBNG leakage and ventilation processes are investigated by CFD simulation. The hazardous gas cloud volume is selected to quantitatively describe the risk. For the leakage process, the evolution of the hazardous gas cloud under different leakage direction, leakage diameter and HBR conditions is analysed. For the ventilation process, the effects of variations in leakage mass, HBR and ventilation conditions on the efficiency of hazardous gas exhaust are mainly analysed. The analysed results are of reference value for the emergency response to HBNG leakage accidents. The nomenclature used is provided in table 1.

## 2. Methods

### 2.1. Physical model

**Figure 1.**
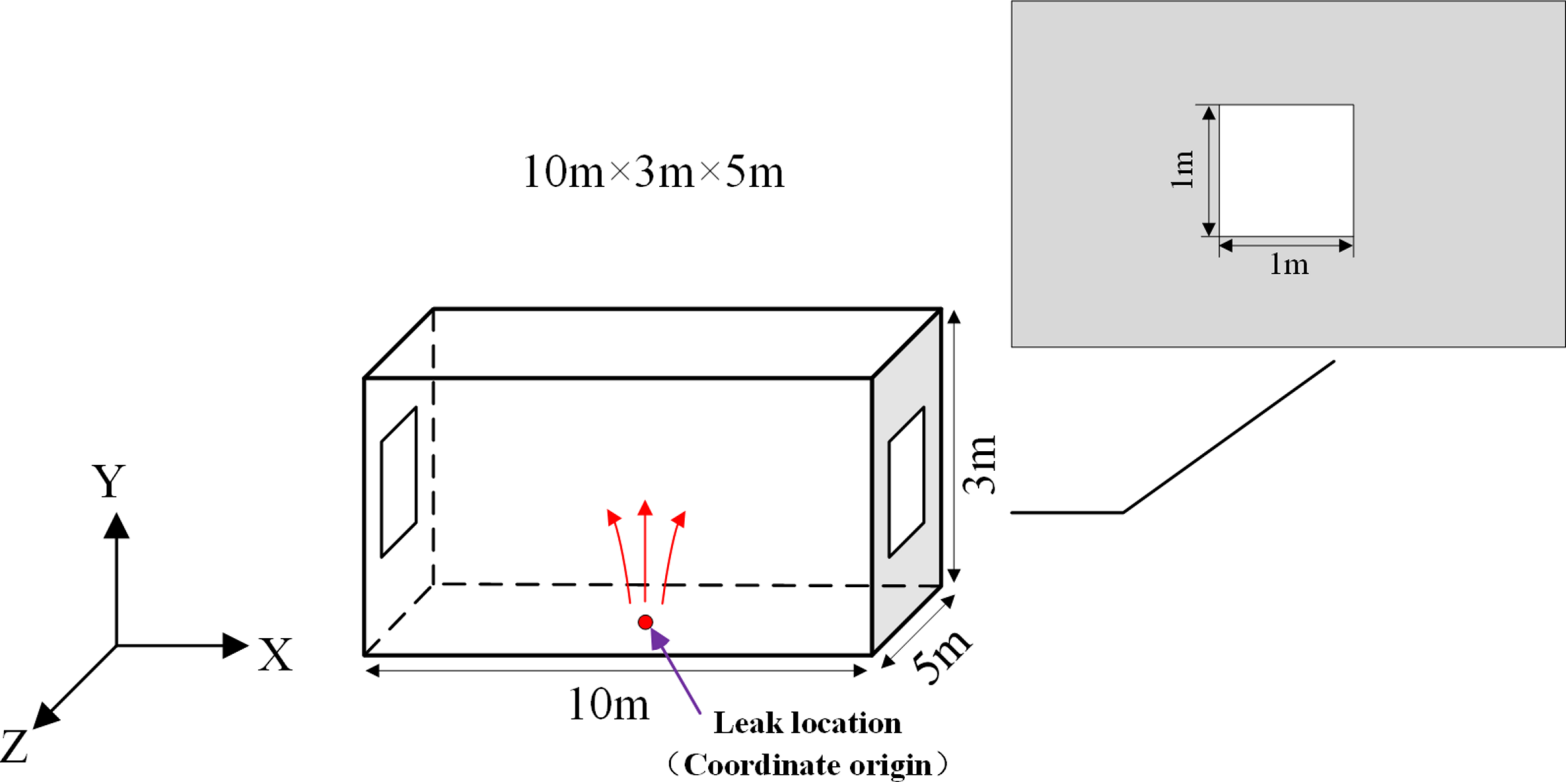
Schematic diagram of the physical model.

A simplified rectangular space is selected as the physical model shown in figure 1 for this work. Its length in *X*, *Y* and *Z* directions is 10 m, 3 m and 5 m, respectively. The volume of the entire space is 150 m^3^. A right-handed coordinate system was chosen for description. The coordinate origin of the system is the centre of the floor in the room, which is exactly where the leakage source is located. The windows are on the *YZ* plane (*X* = 5 m and *X* = −5 m) and have dimensions of 1 m × 1 m. The leakage location is a 10 mm diameter circular hole. It can equate to a low-pressure leakage source in a confined space.

### 2.2. Governing equations and turbulence model

In order to construct the CFD model, some necessary simplifications and assumptions on the physical phenomena of gas flow need to be applied.

(i) Pressure fluctuation in the pipeline system is ignored during leakage. The leakage mass flow rate is constant. (ii) In order to reflect the phenomenon of stratification due to density difference, all gas components are defined as compressible. (iii) H_2_ and CH_4_ do not react chemically during the whole process. (iv) Due to the random layout of real obstacles, other facilities in the space are ignored. (v) Fluid thermal conductivity is isotropic and follows Fourier’s law.

Based on above simplifications and assumptions, conservation equations for gas flow in this work contain the conservation of mass, momentum, energy and component.

The mass conservation (continuity) equation is shown in equation (2.1):


(2.1) $$\frac{\partial \rho }{\partial t}+\nabla \cdot \left(\rho U\right)=0.$$

The momentum conservation equation is shown in equation (2.2):


(2.2) $$\frac{\partial \left(\rho U\right)}{\partial t}+\nabla \cdot \left(\rho UU\right)=-\nabla p+\nabla \cdot \tau +f_{b}.$$

For compressible flow, *τ* is shown in equation (2.3):


(2.3) $$\tau =\mu \left(\nabla U+\nabla U^{T}\right)-\frac{2}{3}\mu \left(\nabla \cdot U\right)I.$$

The component conservation equation is shown in equation (2.4):


(2.4) $$\frac{\partial \left(\rho \omega_{i}\right)}{\partial t}+\nabla \cdot \left(\rho U\omega_{i}\right)=\nabla \cdot \left(D_{i}+\frac{\mu_{t}}{Sc_{t}}\right)\nabla \omega_{i}+R_{i}.$$

The energy conservation equation is shown in equation (2.5) [20]:


(2.5) $$\frac{\partial \left(\rho E\right)}{\partial t}+\nabla \cdot \left(\rho UE\right)=\nabla \cdot \left(\lambda \nabla T\right)+\nabla \cdot \left(pU\right)+\nabla \cdot \left(\tau \cdot U\right)+f_{b}\cdot U+{\overset{˙}{q}}_{v}.$$

There is no internal heat source and no chemical reaction in this study. Therefore, *R_i_* = 0, ${\overset{˙}{q}}_{v}$= 0.

In this work, low-pressure leakage and ventilation processes are mainly investigated. The Reynolds number of gas flow is quite low after momentum dissipation. For this reason, turbulence models for high Reynolds number are not applicable for this work. The shear stress transport (SST) *k−ω* model is selected because of its availability for low Reynolds number flow. The SST *k−ω* model is widely applied because it combines the advantages of the *k−ω* model for near-wall treatment and the *k−ϵ* model for boundary layer edge. Furthermore, the effect of the buoyancy term is considered for this work:


(2.6) $$\frac{\partial \left(\rho k\right)}{\partial t}+\frac{\partial \left(\rho ku_{i}\right)}{\partial x_{i}}=\frac{\partial }{\partial x_{j}}\left[\left(\mu +\frac{\mu_{t}}{\delta_{k}}\right)\frac{\partial k}{\partial x_{j}}\right]+G_{k}+G_{b}-Y_{k},$$

(2.7) $$\frac{\partial \left(\rho \omega \right)}{\partial t}+\frac{\partial \left(\rho \omega u_{i}\right)}{\partial x_{i}}=\frac{\partial }{\partial x_{j}}\left[\left(\mu +\frac{\mu_{t}}{\delta_{\omega }}\right)\frac{\partial \omega }{\partial x_{j}}\right]+G_{\omega }+G_{\omega b}-Y_{\omega }.$$

### 2.3. Boundary and initial conditions

This work involves leakage and ventilation processes. Different boundary conditions should be set for different processes. The details are presented in table 2.

**Table 2.** Boundary conditions of different processes.

boundary name	boundary leakage process	condition type ventilation process
leak hole	mass flow inlet	no-slip wall
walls	no-slip wall	no-slip wall
ground	no-slip wall	no-slip wall
windows	no-slip wall	pressure outlet

For the leakage process, the flow domain is filled with air, and there is no CH_4_ and H_2_ distribution. The temperature is uniformly distributed at 298 K, and the initial gauge pressure is 0 Pa (absolute pressure = 101 325 Pa). The densities of CH_4_ and H_2_ are 0.6681 and 0.0819 kg m^−3^.

The ventilation process initializes in a different way compared with the leakage process. When the residents detect a leakage, they will open a window to exhaust the leaked gas. The moment of opening the windows is a sign that the ventilation process starts. The moment of opening the window is when the ventilation process begins. Therefore, the final state of leakage is the initial state for ventilation. The variable fields obtained from the leakage simulation are interpolated into the flow domain.

### 2.4. The definition of hazardous gas cloud

In this study, the main analysis is conducted for a gas cloud that reaches the explosive limit range. The explosive limit range includes lower explosive limit percentage (LEL%) and upper explosive limit percentage (UEL%). HBNG is multi-component flammable mixture gas. The explosion limit can be calculated according to the volume fraction weighted average method as shown by equation (2.8). The calculation results under different HBRs are shown in table 3.

**Table 3.** Results under different HBRs.

HBR (%)	LEL (%)	UEL (%)
0	4.90	15.00
10	4.81	21.00
20	4.72	27.00
30	4.63	33.00
50	4.45	45.00
70	4.27	57.00
100	4.00	75.00


(2.8) $$L=\frac{100}{\sum_{i=1}^{n}\frac{V_{i}}{L_{i}}}.$$

When the volume fraction of combustible gas is below the LEL or above the UEL, it is indeed not explosive. However, when the volume fraction is above the UEL, it may fall back into the explosive range under the influence of various factors. Therefore, a state above the UEL cannot be considered safe. In this study, the portion of gas volume fraction above the LEL is considered a hazardous gas cloud.

The method of calculating hazardous gas cloud volume is determined based on the characteristics of CFD numerical simulation. The calculation method is shown as equation (2.9), where *V*_d_ is the volume of the hazardous gas cloud, *c_i_* is the gas volume fraction of the cell with NO.*i*, and *V_i_* is the volume of the cell with NO.*i*. First, filter out all the grids with volume fractions higher than the LEL in the flow field. Then, sum up the volume values of these grids. An approximation of the hazardous gas cloud volume can be obtained:


(2.9) $$V_{d}=\sum_{i=1}^{n}V_{i}\left(c_{i}\geq \text{LEL}\right).$$

### 2.5. Work conditions

**Figure 2.**
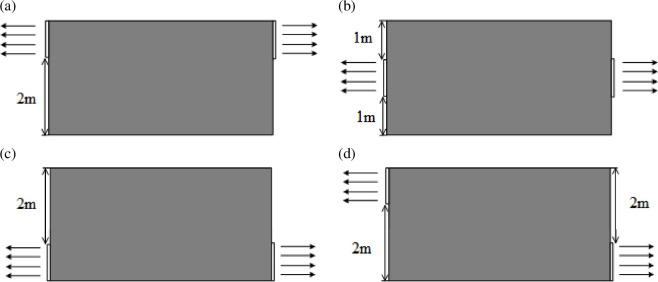
Different ventilation conditions in this work.

The arrangement of windows in real scenarios is variable. Especially for kitchens in small restaurants, most of them have been remodelled. Different ventilation conditions according to window locations are defined, as shown in figure 2. The arrows represent the direction of leaked gas flow. The windows are all 1 m × 1 m consistent with the size in figure 1.

In order to reflect differences in physical properties of gas mixtures, HBRs are selected as 0, 10, 20, 30, 50, 70 and 100%. The window status is closed for the gas leakage process and open for the ventilation process. The pressure of domestic natural gas is 2000 Pa. The mass flow rate is referred to the prior study by Li *et al*. with dimension modifications [9]. For HBR = 0%, the mass flow rate is 0.003006 kg s^−1^ with 10 mm leakage diameter. The corresponding volume flow rate is about 0.0045 m^3^ s^−1^. Most gas cooker hoses in China have a diameter of 9.5 mm. Therefore, the leakage aperture for leakage work conditions is 0–9.5 mm. The low-pressure conditions of this study result in subsonic flow at the leakage location. According to the leakage model, the leakage volume flow rate is proportional to the leakage area under constant pressure conditions [21]. In this study, the volume flow rate is considered to be constant for the same leakage diameter. Volume flow rates for different leakage diameters are converted to 10 mm diameter in the study of Li *et al*. [9]. They are consistent with the leakage intensity of urban low-pressure pipelines under operation pressure.

**Figure 3.**
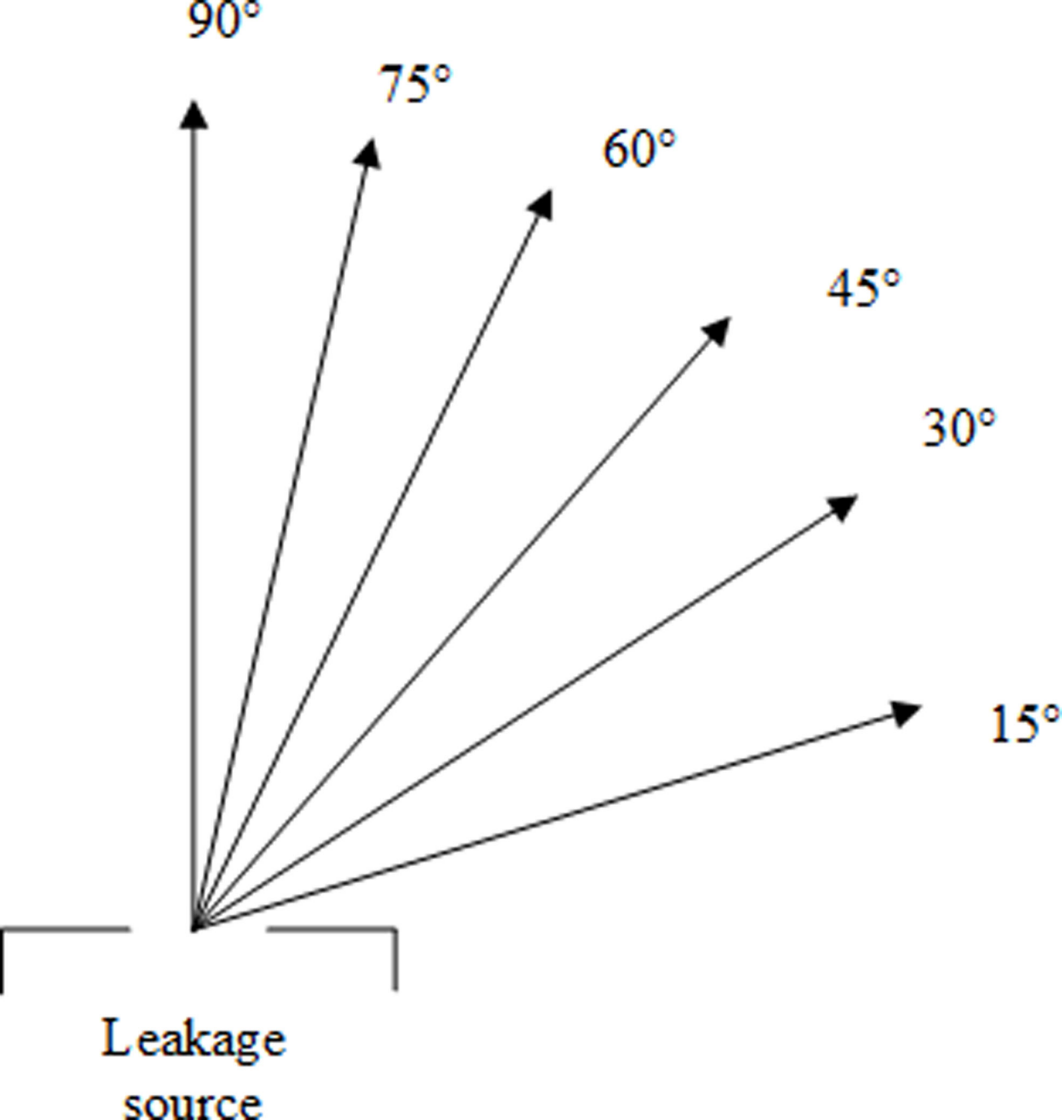
The diagram of different leakage directions.

The penetration rate of gas alarms in Chinese urban areas is at a low level, which is less than 10% [22]. Gas leakage accidents are often not dealt with rapidly. For this reason, the length of the leakage stage simulation is set to over 3600 s in this study. The work conditions for the leakage process and the ventilation process are shown in tables 4 and 5. The leakage degree represents the direction of gas jet as shown in figure 3.

**Table 4.** Work conditions for leakage processes.

no.	HBR (%)	mass flow (kg s^−1^)	leakage diameter (mm)	leakage degree (°)
case 1	0	0.002796	9.5	90
case 2	10	0.002506	9.5	90
case 3	20	0.002305	9.5	90
case 4	30	0.00206	9.5	90
case 5	50	0.001569	9.5	90
case 6	70	0.001078	9.5	90
case 7	100	0.0003428	9.5	90
case 8	0	0.001691	7.5	90
case 9	10	0.001543	7.5	90
case 10	20	0.001394	7.5	90
case 11	30	0.001246	7.5	90
case 12	50	0.000949	7.5	90
case 13	70	0.000652	7.5	90
case 14	100	0.000207	7.5	90
case 15	0	0.000909	5.5	90
case 16	10	0.00083	5.5	90
case 17	20	0.00075	5.5	90
case 18	30	0.00067	5.5	90
case 19	50	0.00051	5.5	90
case 20	70	0.000351	5.5	90
case 21	100	0.000111	5.5	90
case 22	10	0.002506	9.5	15
case 23	10	0.002506	9.5	30
case 24	10	0.002506	9.5	45
case 25	10	0.002506	9.5	60
case 26	10	0.002506	9.5	75
case 27	10	0.001543	7.5	15
case 28	10	0.001543	7.5	30
case 29	10	0.001543	7.5	45
case 30	10	0.001543	7.5	60
case 31	10	0.001543	7.5	75
case 32	10	0.00083	5.5	15
case 33	10	0.00083	5.5	30
case 34	10	0.00083	5.5	45
case 35	10	0.00083	5.5	60
case 36	10	0.00083	5.5	75

**Table 5.** Work conditions for ventilation processes.

no.	HBR (%)	mass flow (kg s^−1^)	window status	ventilation
case 1	0	10.07	open	high
case 2	0	10.07	open	centre
case 3	0	10.07	open	low
case 4	0	10.07	open	mix
case 5	10	9.18	open	high
case 6	10	9.18	open	centre
case 7	10	9.18	open	low
case 8	10	9.18	open	mix
case 9	20	8.30	open	high
case 10	20	8.30	open	centre
case 11	20	8.30	open	low
case 12	20	8.30	open	mix
case 13	30	7.42	open	high
case 14	30	7.42	open	centre
case 15	30	7.42	open	low
case 16	30	7.42	open	mix
case 17	50	5.65	open	high
case 18	50	5.65	open	centre
case 19	50	5.65	open	low
case 20	50	5.65	open	mix
case 21	70	3.88	open	high
case 22	70	3.88	open	centre
case 23	70	3.88	open	low
case 24	70	3.88	open	mix
case 25	100	1.23	open	high
case 26	100	1.23	open	centre
case 27	100	1.23	open	low
case 28	100	1.23	open	mix
case 29	10	13.77	open	high
case 30	10	13.77	open	centre
case 31	10	13.77	open	low
case 32	10	13.77	open	mix
case 33	10	18.36	open	high
case 34	10	18.36	open	centre
case 35	10	18.36	open	low
case 36	10	18.36	open	mix

In this work, the pressure implicit with splitting of operators (PISO) algorithm is applied to deal with the pressure−velocity coupling. The details of the discrete format for various physical variables are shown in table 6. To ensure solution stability, the time step is set with Courant number < 5. The fixed time step is applied as 0.05 s and the maximum iteration per time step is set as 200.

**Table 6.** Solution strategies and methods.

item	method
pressure–velocity coupling method	PISO
gradient	least squares cell-based
pressure	second-order upwind
energy	second-order upwind
momentum	second-order upwind
species	second-order upwind
specific dissipation rate	second-order upwind
turbulent kinetic energy	second-order upwind

### 2.6. Grid independence and model validation

**Figure 4.**
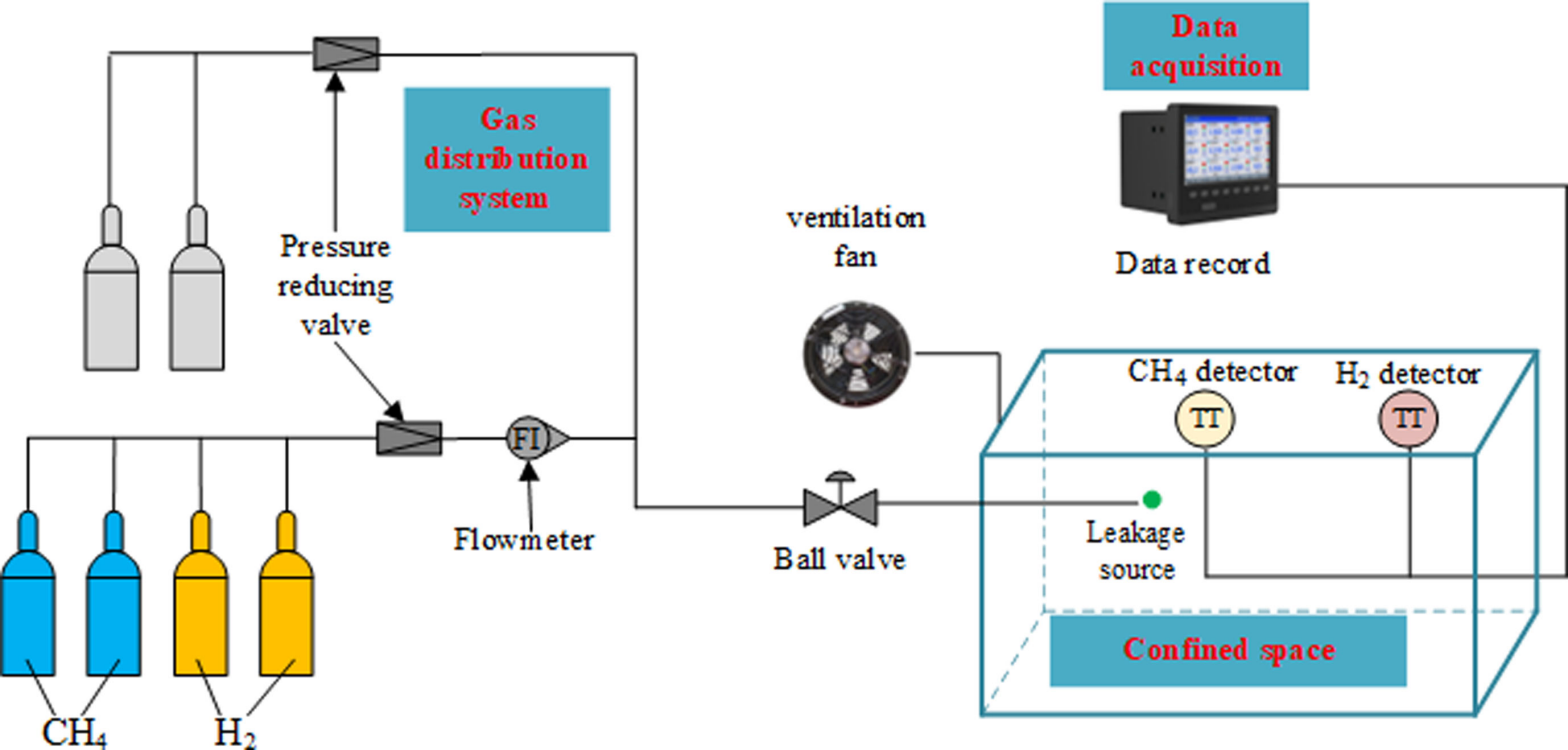
Experiment system.

**Figure 5.**
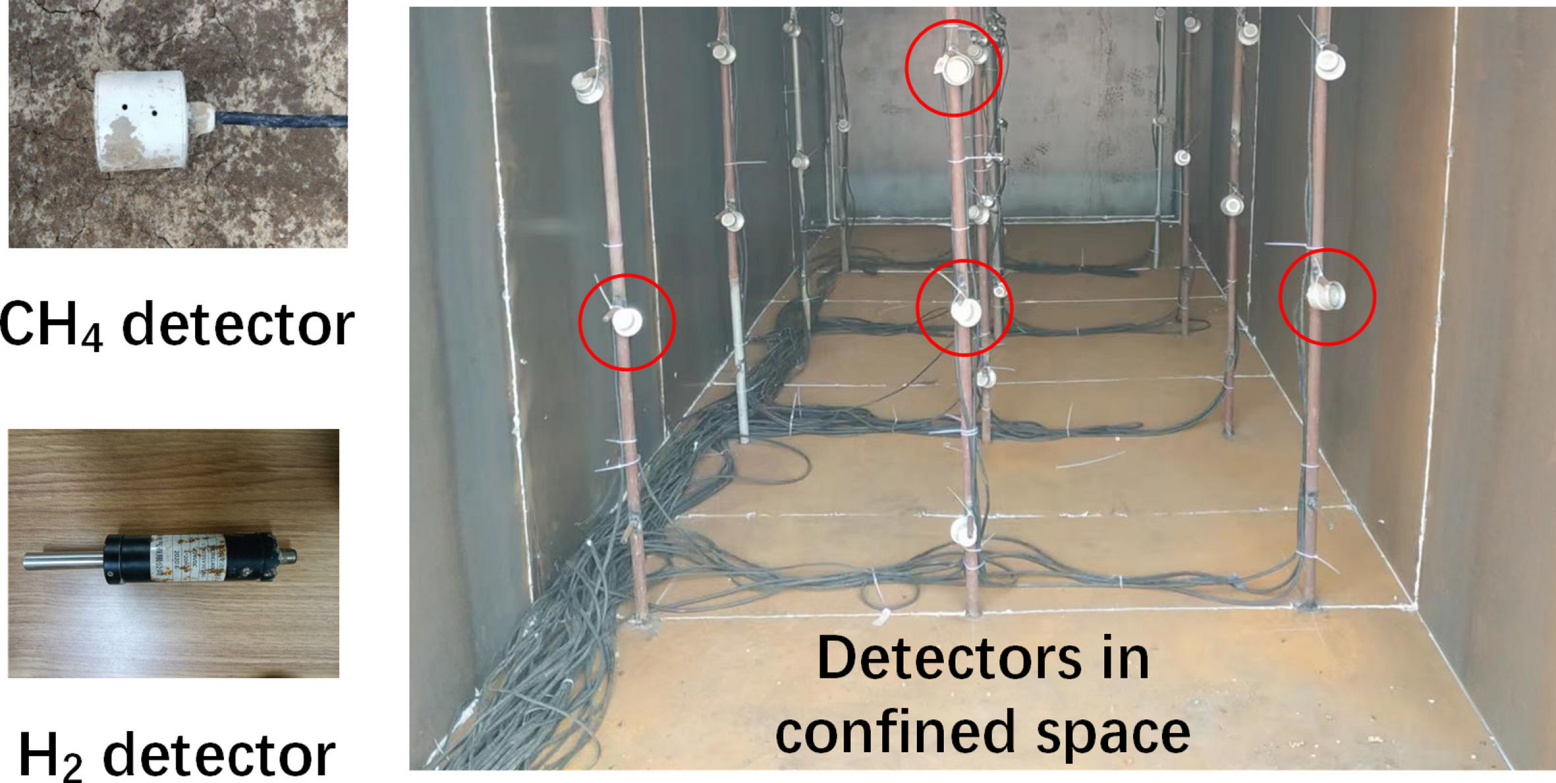
Gas detectors in confined space.

In order to validate the reliability of the CFD model, some experiments on leakage process conditions were conducted using the experiment system shown in figure 4. Photographs of the gas detectors and the interior of confined spaces are shown in figure 5. The dimensions of the confined space in the experimental system are not the same as those of the physical model in the CFD simulation. The confined space in the experiment system has dimensions of 6.7 m × 3.3 m × 2 m. The ratio of the confined dimensions in the CFD model to those in the experiment is approximately 1.5. Therefore, in order to keep the distribution pattern of the leaked gas the same, the leakage flow rate in the experiment needs to be determined based on the similarity criterion. Liu *et al*. have conducted gas leakage experiments based on similarity of Froude number [23]. The similarity ratios of simulation and experiment are shown in table 7. The model validation was carried out with the working condition of HBR = 20%. At this point, the leakage flow rate of the simulation model is 0.002506 kg s^−1^. Based on the similarity ratio conversion in table 8, the corresponding leakage flow rate for the experiment is 0.000909 kg s^−1^. The heights of the monitoring points are also converted according to similarity ratios. The specific information is shown in table 8. After this conversion, the CFD simulation result can be comparable with the experiment result. It means that the similarity ratio of gas concentration at the monitoring point is 1.

**Table 7.** Similarity ratio of variables (simulation model:experiment model)

comparison variable	similarity ratio
geometry dimensions (m)	$\frac{3}{2}$
leakage rate (kg s^−1^)	${\left(\frac{3}{2}\right)}^{5/2}$
monitoring height (m)	$\frac{3}{2}$
gas concentration	1

**Table 8.** Simulation and experimental parameters for validated working condition.

comparison variable	experiment	simulation
geometry dimensions (m)	6.7 × 3.3 × 2	10 × 5 × 3
leakage rate (kg s^−1^)	0.000909	0.002506
monitoring point (m)	(0, 0.5, 0)	(0, 0.75, 0)

**Figure 6.**
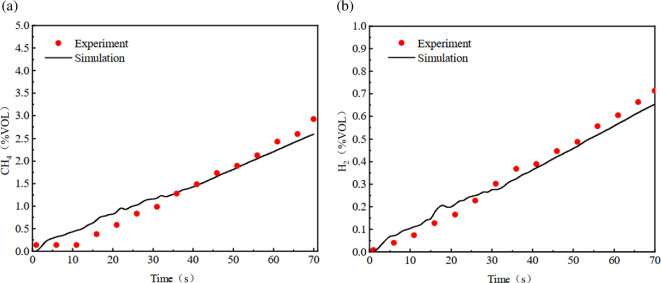
Comparison of numerical simulation and experiment results.

CFD models equivalent to the experiments are built with the numerical method designed in this study. The result of leaked gas concentration variation at monitoring point is shown in figure 6*a*,*b*. It can be noticed that the simulated and experiment volume fractions of hydrogen and methane are close to each other at the initial stage of leakage. The experiment results are slightly lower than the simulation results at the initial stage of leakage, and then gradually exceed the simulation results. The average error does not exceed 13.1%. It can be considered that the numerical method and solution strategy are reasonable and reliable.

**Figure 7.**
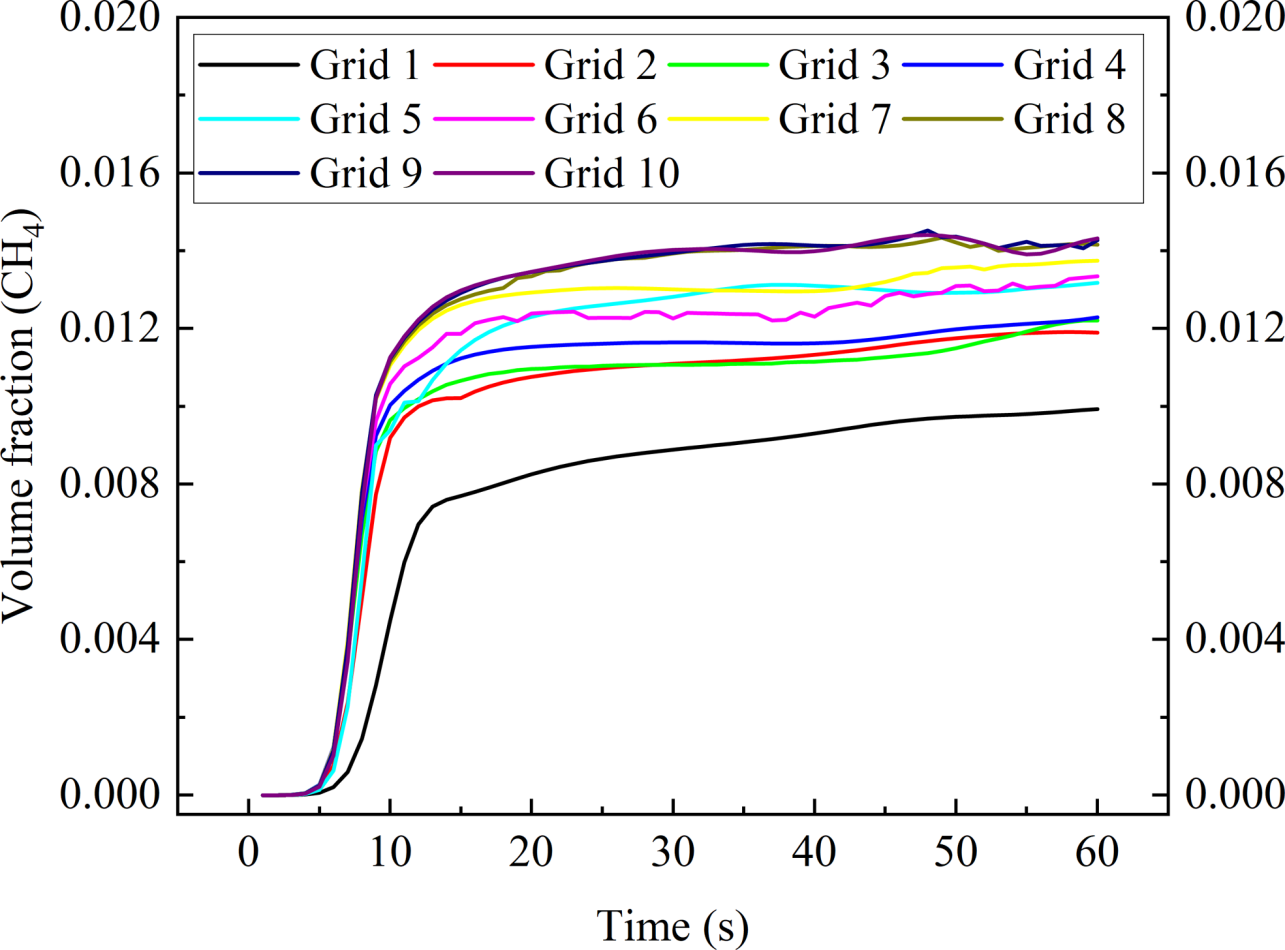
CH_4_ volume fraction variation at monitoring point.

**Figure 8.**
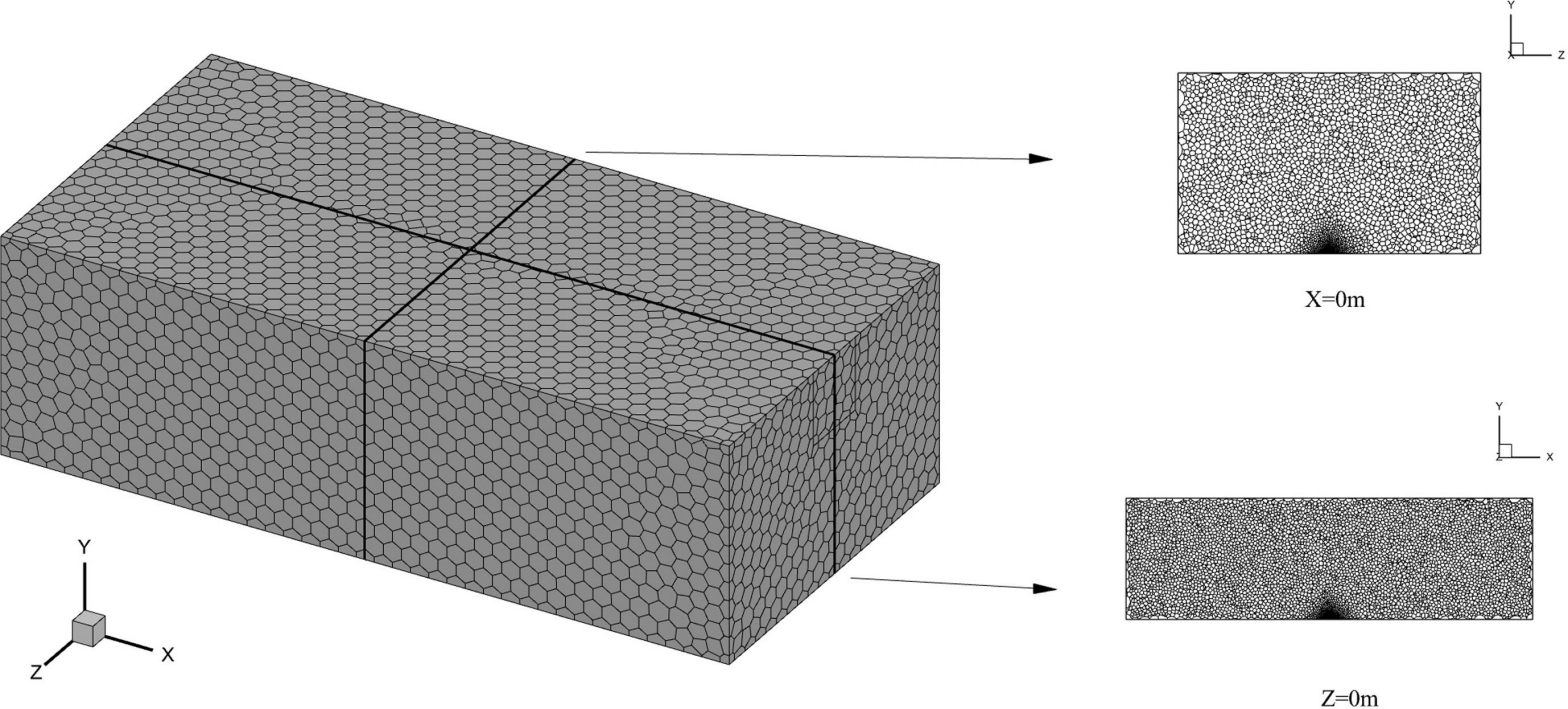
Generated grid for numerical simulation.

In this study, polyhedral grids are applied for grid independence. Cell number information is shown in table 9. Case 1 in table 4 is selected for comparison. The coordinates of the monitoring point are selected as 0 m, 2.7 m, 0 m, which are located directly above the leakage source. Figure 7 shows the variation of methane volume fraction in the initial stage (0–60 s) at the monitoring point. It can be found that as the number of grid cells rises, the volume fraction under each moment also rises. When the number of grid cells reaches 321 028, the variation is no longer significant. Therefore, the grid with 321 028 cells is applied for this work in order to increase computation efficiency. The final grid generated is shown in figure 8, which corresponds to the work condition where window 1 and window 2 are located in high position. As the window position varies, the number of generated grid cells changes slightly. This fluctuation does not have a significant influence on the results because the number of grid cells remains floating at about 320 000. The maximum and minimum sizes of the grid at this point are 1 and 90 mm, respectively. The SST model requires a high resolution of the near-wall grid; therefore, five prism layer grids with long aspect ratios are assigned near walls. By this measure, it ensures that *Y*+ is less than 5 at most of the walls.

**Table 9.** The cell numbers of different grids.

item	cell number	item	cell number
grid 1	44 186	grid 6	120 083
grid 2	57 258	grid 7	202 117
grid 3	82 226	grid 8	321 028
grid 4	90 870	grid 9	431 403
grid 5	100 038	grid 10	615 468

## 3. Results and discussion

### 3.1. Hazardous gas cloud evolution under leakage process

#### 3.1.1. Effect of leakage direction and diameter on formation of hazardous gas cloud

**Figure 9.**
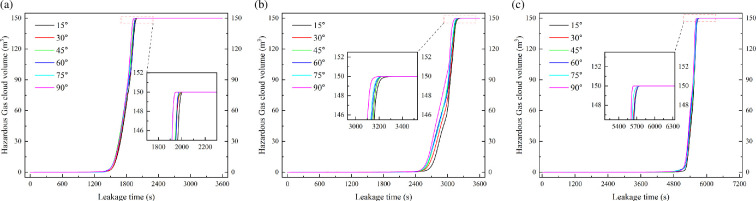
Hazardous gas cloud volume variation under different leakage degree.

The hazardous gas volume variation results are shown in figure 9. It shows the hazardous gas cloud volume variation for leakage diameters of 9.5, 7.5 and 5.5 mm. Hazardous gas cloud volume variations in different leak directions are compared for each leak diameter. The leaked HBNG at this point corresponds to HBR = 10%. It can be found that the hazardous gas cloud volume variation curves under every leakage direction almost overlap. Just slight differences between the volume variation curves can be detected partially. With a leakage direction of 90°, the hazardous gas cloud volume would earlier fill the entire space.

**Figure 10.**
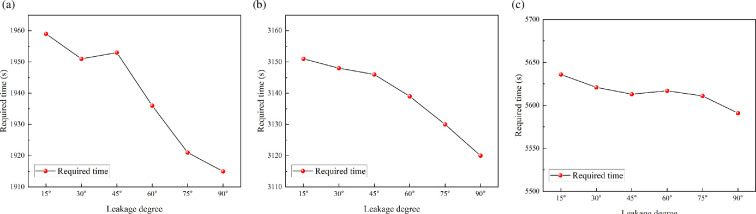
Required time variation under different leakage degree and leakage diameter.

The required time for the hazardous gas cloud fill the entire space is shown in figure 10. It can be found that the required time decreases as the angle rises. The required time is minimum when the leakage direction is vertical. This conclusion is valid for each of the leakage diameters. However, there is little difference in required time under each angle. These time differences are quite short for the accumulation process of hazardous gas cloud, which is less than 5%. Therefore, for engineering applications, it is sufficiently representative to analyse only the vertical up leakage scenario.

**Figure 11.**
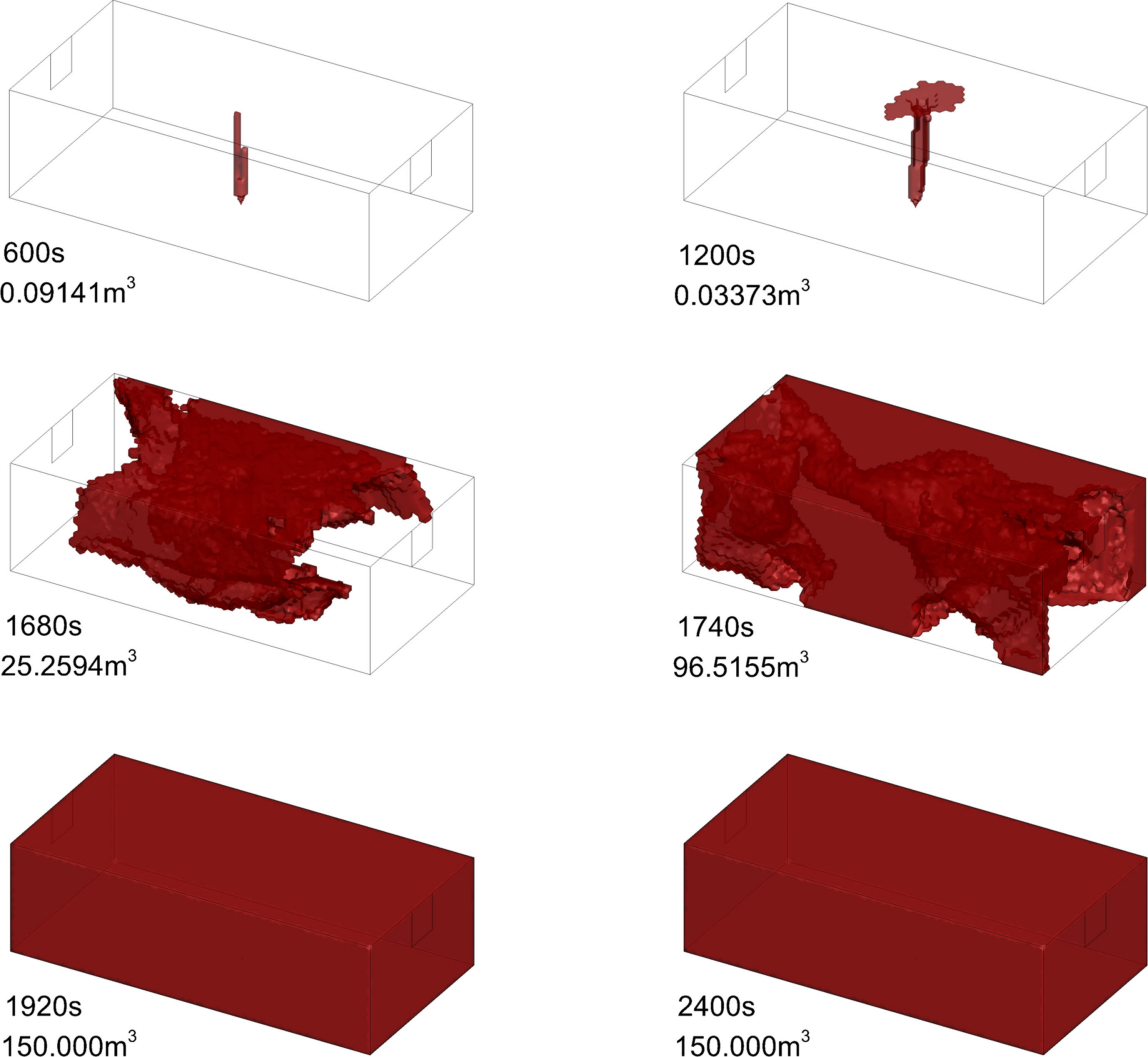
Hazardous gas cloud evolution for 9.5 mm.

For the leakage process, the appearance of hazardous gas clouds shows a delayed character. The hazardous gas cloud volume remains at 0 m^3^ for some time and then rises rapidly to 150 m^3^ after a specific moment. Taking 9.5 mm as an example, the variation of hazardous gas cloud is shown in figure 11. It shows that the hazardous gas cloud does not vary significantly from 600 s to 1200 s but expands rapidly from 1680 s to 1920 s. It is worth noting that the gas distribution inside the hazardous gas cloud is not homogeneous. The leaked gas will show a degree of stratification [10].

**Figure 12.**
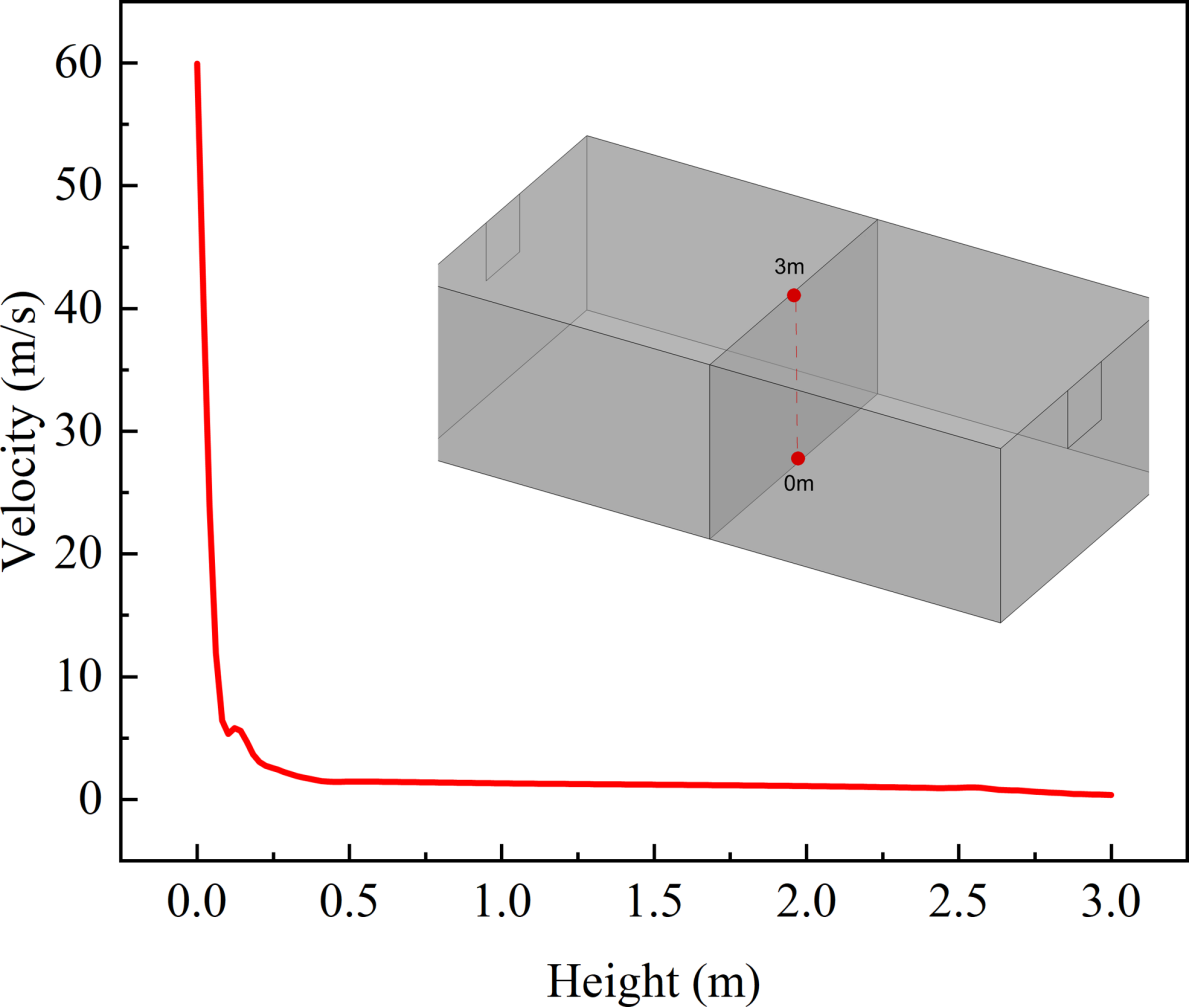
Velocity distribution in *Y* direction.


For the small time differences in figure 10, it can be analysed based on the properties of leakage jet. Froude number is used to describe the ratio of momentum and buoyancy effects of the jet [24]. This dimensionless variable represents the ratio of inertial force to buoyancy force. Leakage with high *Fr* is dominated by the initial momentum of the gas jet. Leakage with low *Fr* is dominated by buoyancy forces. The velocity variation with height above the leakage source is shown in figure 12 for leakage diameter of 9.5 mm. It can be found that the velocity decreases quite rapidly with height. The effect of initial momentum will be eliminated at low height. The leaked gas is mainly buoyancy-driven to form a plume in the far field of leakage. Variations in leakage direction do not lead to differences in this feature. Ultimately, it makes little difference in the required time for hazardous gas cloud filling the space.

(3.1) $$Fr=\frac{U_{exit}}{\sqrt{gd\left(\rho_{\infty }-\rho_{exit}\right)/\rho_{exit}}}.$$

#### 3.1.2. Effect of hydrogen-blended ratio on hazardous gas cloud volume variation

Based on the findings of Sun *et al.*, the increase of HBR shortens both the alarm response time and the time to reach LEL [8]. It can be hypothesized that the variation of hazardous gas cloud volume has a similar variation pattern.

**Figure 13.**
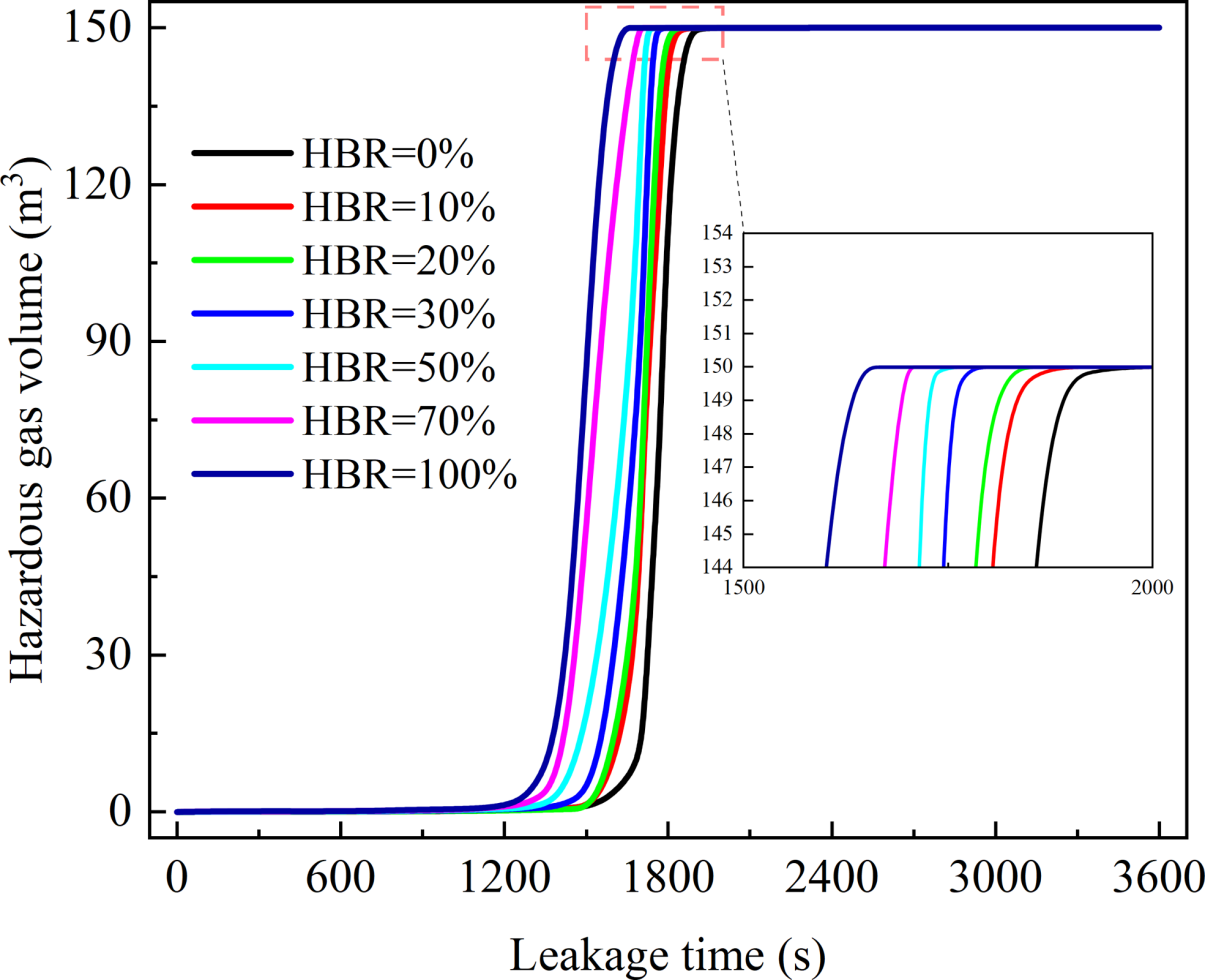
Hazardous gas cloud volume variation under HBRs.

The variation of hazardous gas cloud volume is shown in figure 13 under different HBRs. During this process, the windows are closed. The trend of accumulation process of the hazardous gas cloud is similar for different HBRs. It can be found that the volume is nearly 0 m^3^ for a long time. When leak time is more than 1200 s, the volume shows a rapid increasing trend. With HBR increasing, the moment that hazardous gas volume reaches 150 m^3^ decreases slightly.

**Figure 14.**
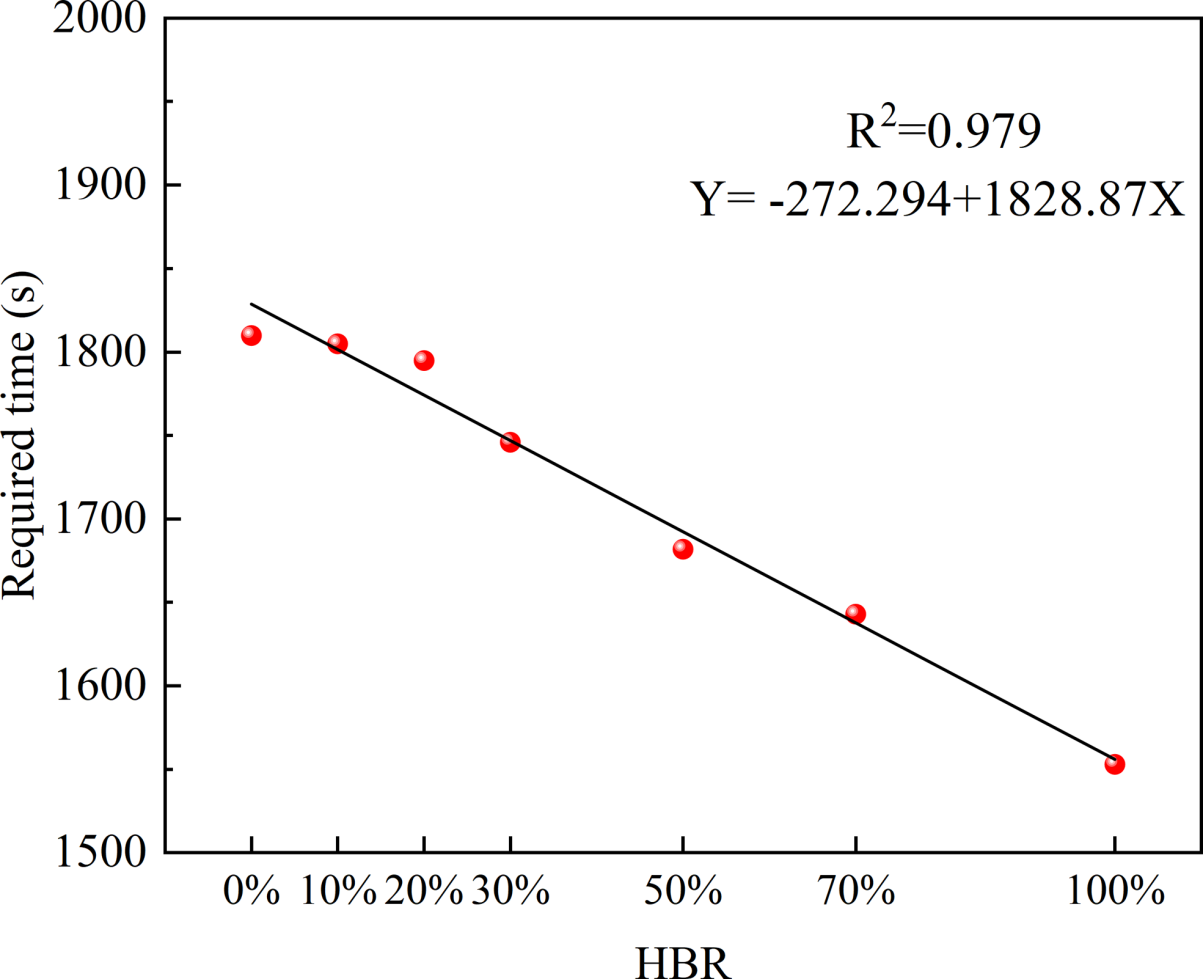
Required time to reach 150 m^3^.

The required time for hazardous gas cloud filling the space is also related to HBR. It decreases as the HBR rises as shown in figure 14. The relationship between required time and HBR showed an approximate linear relationship. It validates the hypothesis presented earlier that an increase in HBR shortens the process of hazardous gas cloud development [8]. But the time difference does not exceed 360 s. With the same leakage volume rate, the risk develops slightly faster for higher HBR, but the overall difference is not significant.

The slight difference of required time for hazardous gas cloud filling the space is mainly due to variation in explosion limit. LELs for CH_4_ and H_2_ are very close. It makes the LEL difference less than 1% under different HBRs as shown in table 3. The evolution of hazardous gas cloud volume is similar. At present, the HBR for engineering applications is now less than 30%. Compared with pure natural gas, the required time difference is limited to 15 s. Variations in LEL do not have a significant influence during the hazardous gas cloud accumulation stage.

#### 3.1.3. Analysis for increasing stage of hazardous gas cloud volume

**Figure 15.**
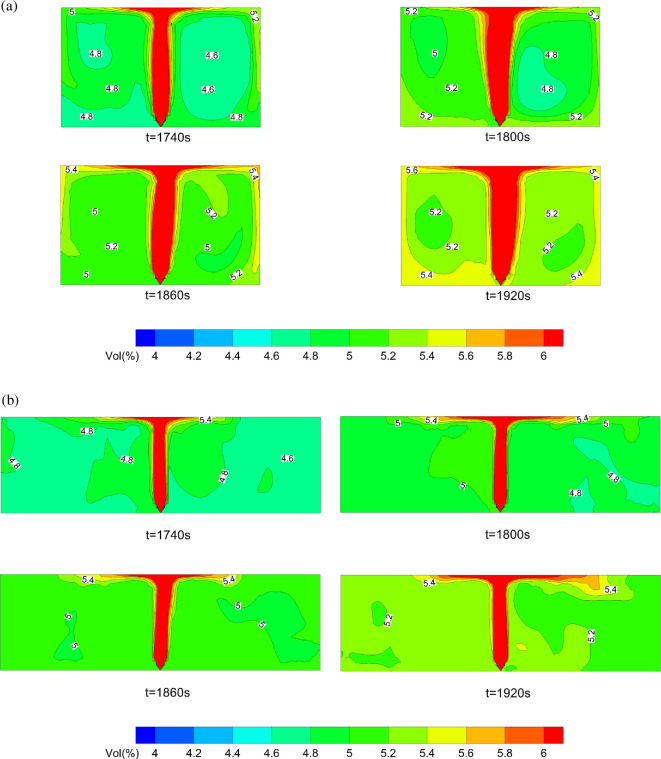
HBNG volume fraction variation at rapid increasing stage.

In the previous section, we note a rapid increase in hazardous gas cloud volume after 1200 s. It can be explained by combining it with the distribution of leaked HBNG. Figure 15 shows leaked HBNG distribution on *X* = 0 m and *Z* = 0 m under HBR = 0%. To facilitate expression, vol(%) is also used to indicate volume fraction. The figure shows that leaked HBNG is relatively uniformly distributed in other areas except for above the leakage source. The concentration gradient of HBNG is quite small.

Next, the variation of vol(%) on the planes is analysed according to chronological orders. First, it needs to be clarified that HBNG with HBR = 0% corresponds to LEL of 4.9%. At 1740 s, vol(%) in most area is close to LEL. At 1860 s, most areas have already exceeded LEL. At 1920 s, vol(%) has already reached LEL. In summary, vol(%) at most locations reaches LEL almost at the same time. Considering the calculation method of hazardous gas cloud volume in this study, this phenomenon will lead to rapid increase.

According to prior studies on gas jets, the larger the initial momentum of the jet, the more significant the entrainment effect on ambient air [10]. In this study, the initial momentum of the gas jet is small and the jet entrainment effect on surrounding air is not obvious. Eventually, the HBNG distribution is more uniform in all areas except above the leakage source.

**Figure 16.**
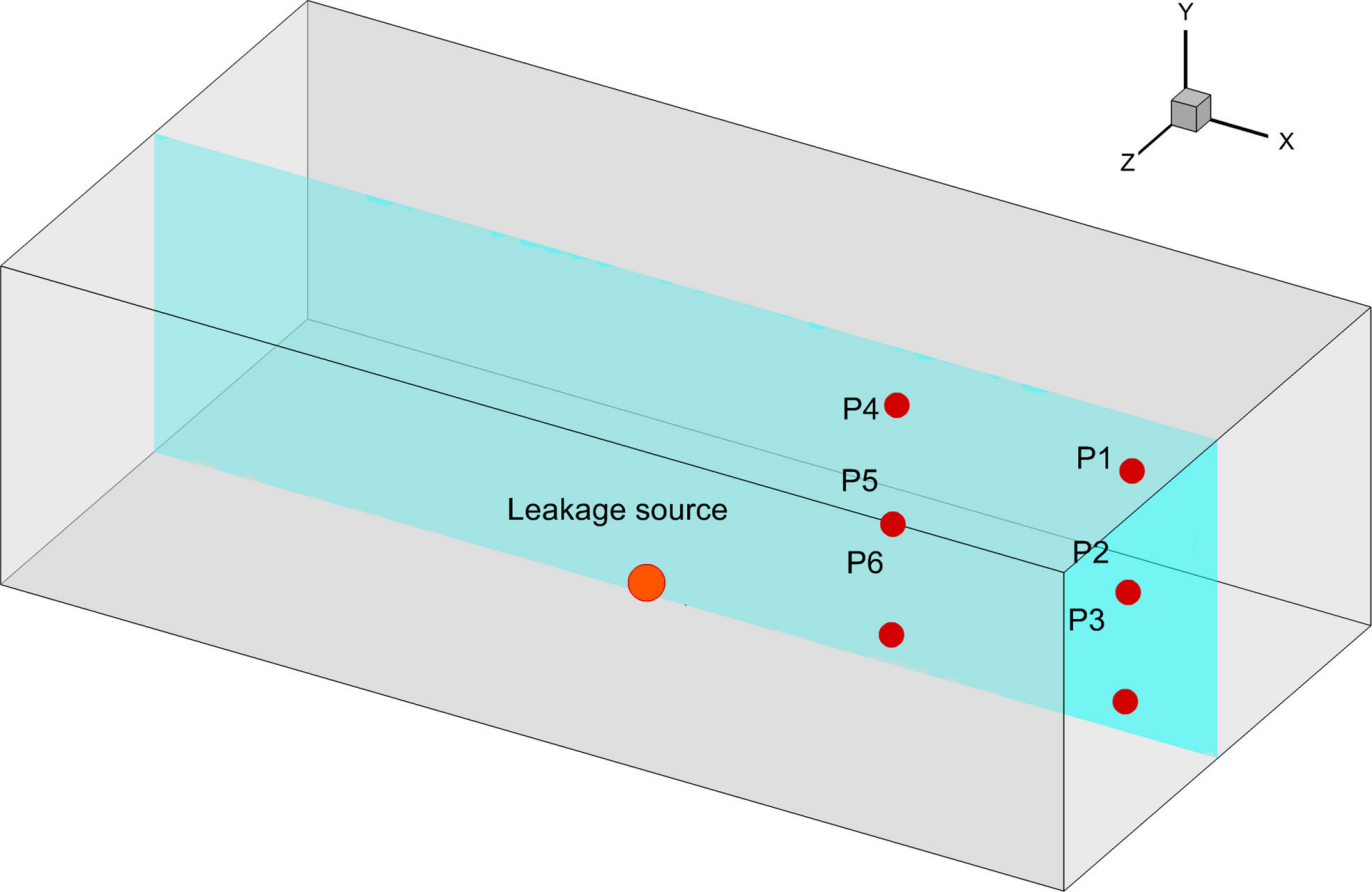
The diagram of leakage source and monitoring points.

**Figure 17.**
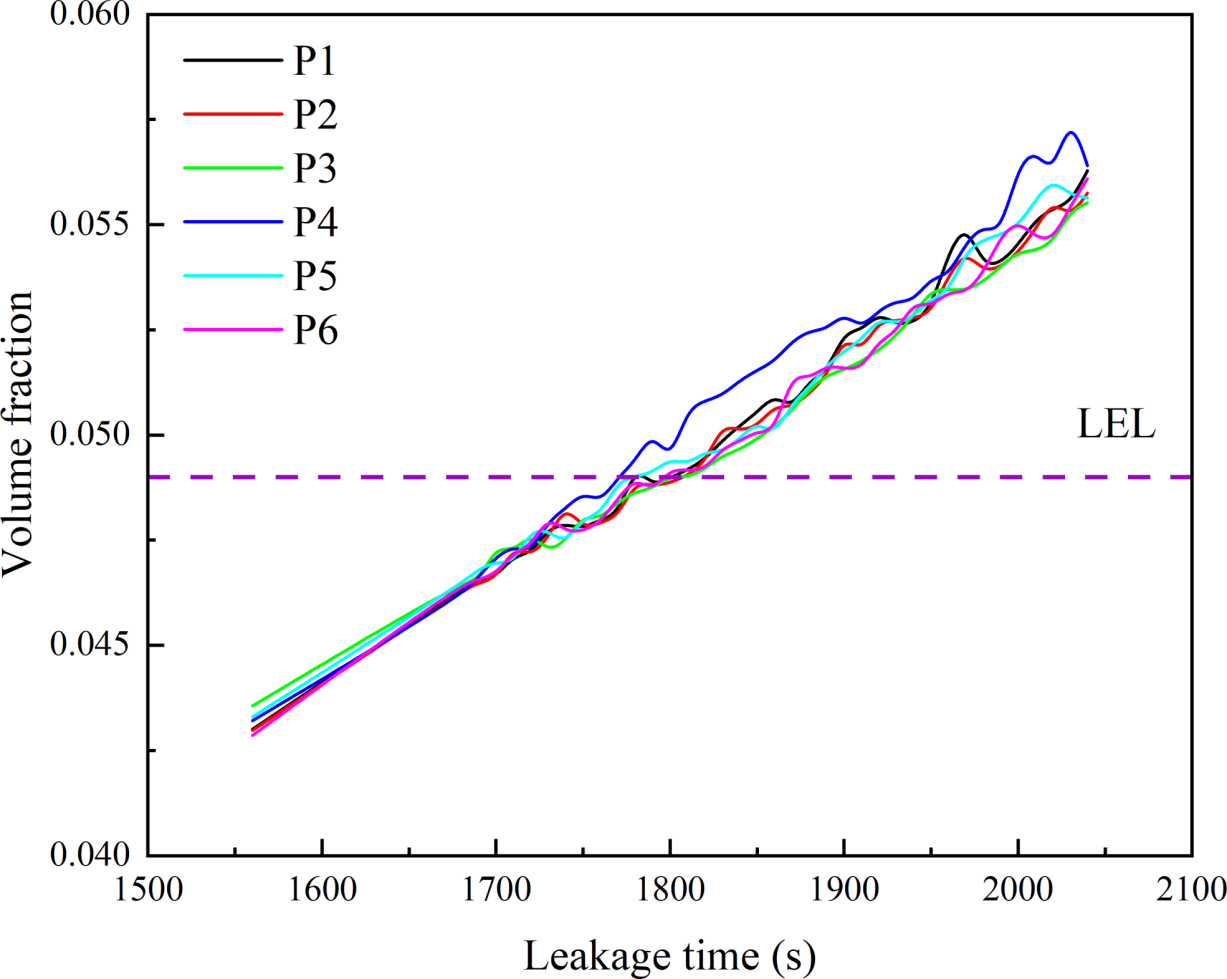
HBNG volume fraction variation at monitoring points.

The uniformity of leaked gas distribution can also be verified by the variation of volume fraction at monitoring points. Six monitoring points are shown in figure 16. These monitoring points are located on the *Z* = 0 m plane at different heights. The specific coordinates are shown in table 10. It can be noted that monitoring points are all located in the far field of leakage location. They cannot be significantly affected by the jet. The variation in volume fraction at monitoring points is shown in figure 17. The volume fraction variation curves are quite close at different heights. The time difference is less than 30 s. The difference in moments when the volume fraction reaches the LEL is also quite minimal. This feature verifies the conclusions in the previous paragraph.

**Table 10.** Coordinates of monitoring points.

monitoring points	*X* (m)	*Y* (m)	*Z* (m)
P1	4.75	2.75	0
P2	4.75	1.5	0
P3	4.75	0.25	0
P4	2.25	2.75	0
P5	2.25	1.5	0
P6	2.25	0.25	0

The distribution of leaked HBNG is tending to uniform except for the near field of the leakage location. The difference in volume fraction at each location is quite small. Therefore, each location reaches LEL at approximately the same time. Ultimately, it produces a rapidly increasing stage in hazardous gas cloud volume.

**Figure 18.**
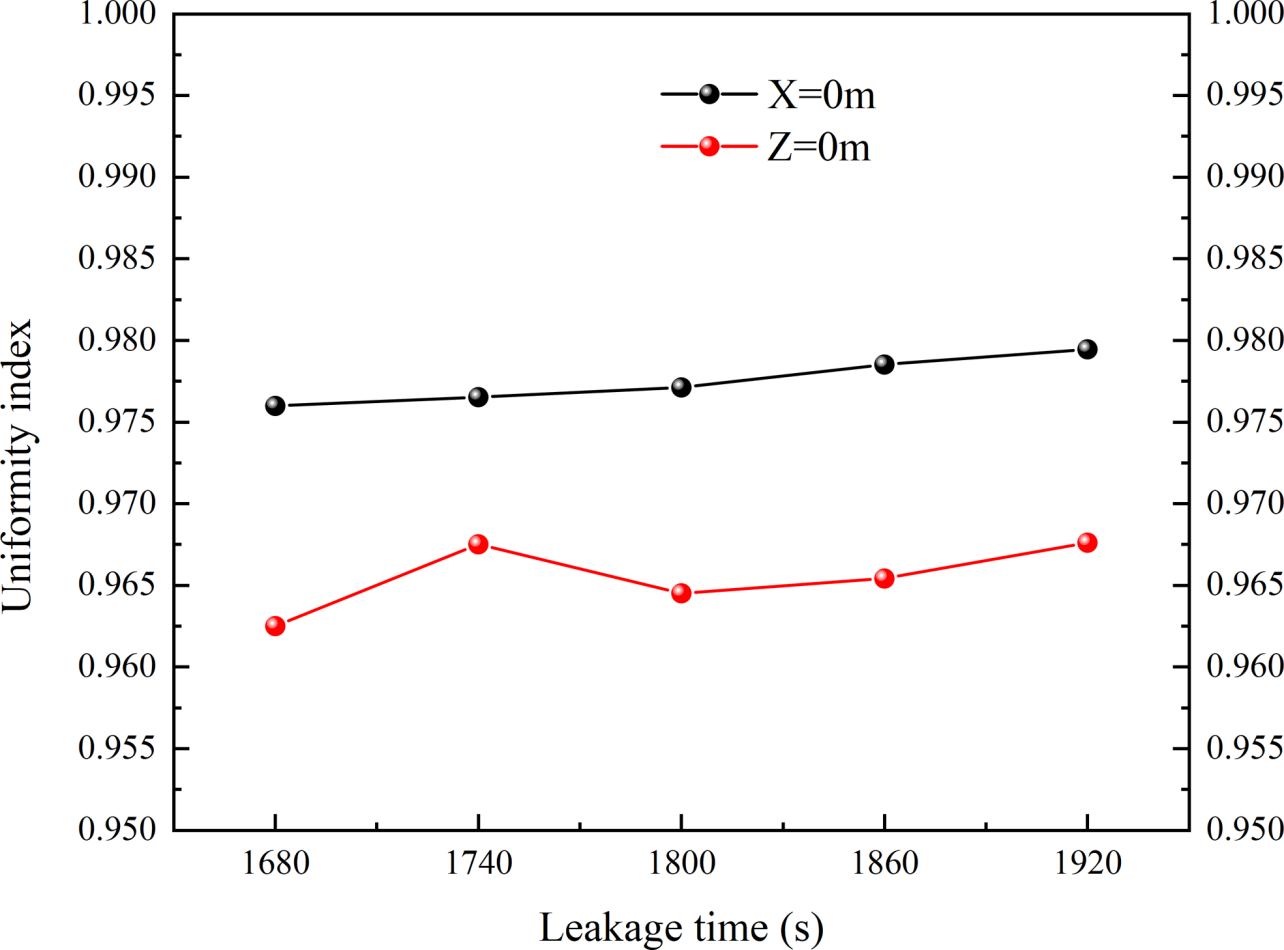
Uniformity index variation from 1680 s to 1920 s.

The uniformity of gas distribution can be expressed quantitatively in dimensionless variables. Equation (3.2) is for the calculation of volume fraction uniformity index:


(3.2) $$\gamma_{a}=1-\frac{\sum_{i=1}^{n}|Vol_{i}-Vol_{a}|A_{i}}{2|Vol_{a}|\sum_{i=1}^{n}A_{i}},$$

where *Vol* represents volume fraction of HBNG, *A_i_* represents area of every cell, *Vol_i_* represents volume fraction of every cell, nd *Vol_a_* represents average volume fraction of all cells. So *γ_a_* closer to 1 represents the higher uniformity of HBNG *Vol*. Figure 18 shows the uniformity index variation of *X* = 0 m and *Z* = 0 m corresponding to figure 15. Its value ranges between 0.96 and 0.98, which is very close to 1. It indicates quantitatively that the distribution of leaked HBNG is uniform. All locations trend to reach LEL at approximately the same time. Eventually, it leads to the rapid increase phase for hazardous gas cloud volume.

### 3.2. Hazardous gas cloud evolution for ventilation process

#### 3.2.1. Effect of total leakage mass and ventilation condition on ventilation process

For the simulation of the ventilation process, gas concentration data at 3600 s of leakage are used to initialize the flow field. The reason for choosing this moment is that the hazardous gas cloud is 150 m^3^ in volume at this time. This means the entire confined space is filled with hazardous gas cloud. It is convenient to analyse the evolution of the gas cloud volume.

**Figure 19.**
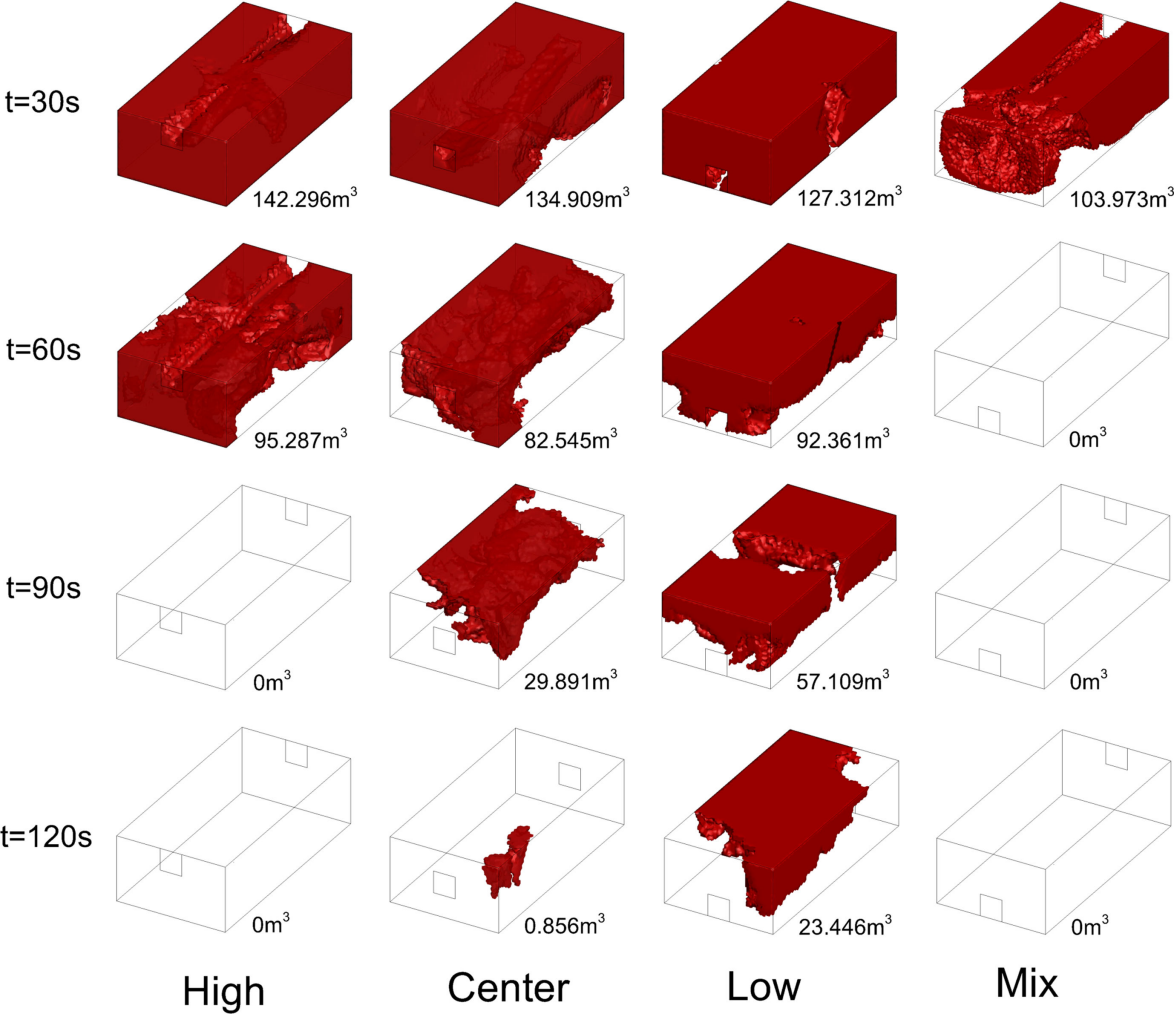
Hazardous gas cloud variation for HBR = 10%.

Figure 19 shows the variation of hazardous gas cloud shape under different ventilation conditions. The total mass of leaked gas is 9.18 kg with HBR = 10%. The time for hazardous gas cloud volume to decrease to 0 m^3^ is taken as a criterion for judging ventilation performance. The hazardous gas cloud volume decreases to 0 m^3^ considering the hazardous gas to be completely exhausted. It can be found that the rankings of required time are low > centre > high > mix. The less time it takes for hazardous gas volume decrease to 0 m^3^ represents better ventilation. The higher vent location performs better when both vents are the same height, whereas the mix condition with one high and one low vent performs best.

**Figure 20.**
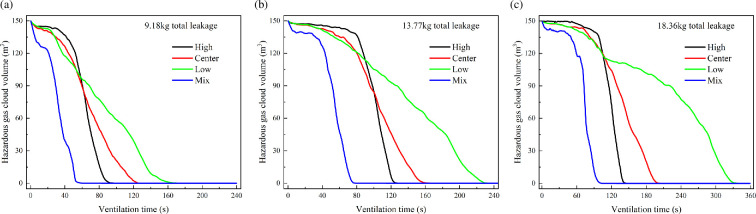
Hazardous gas cloud volume variation under different total leakage mass.

**Figure 21.**
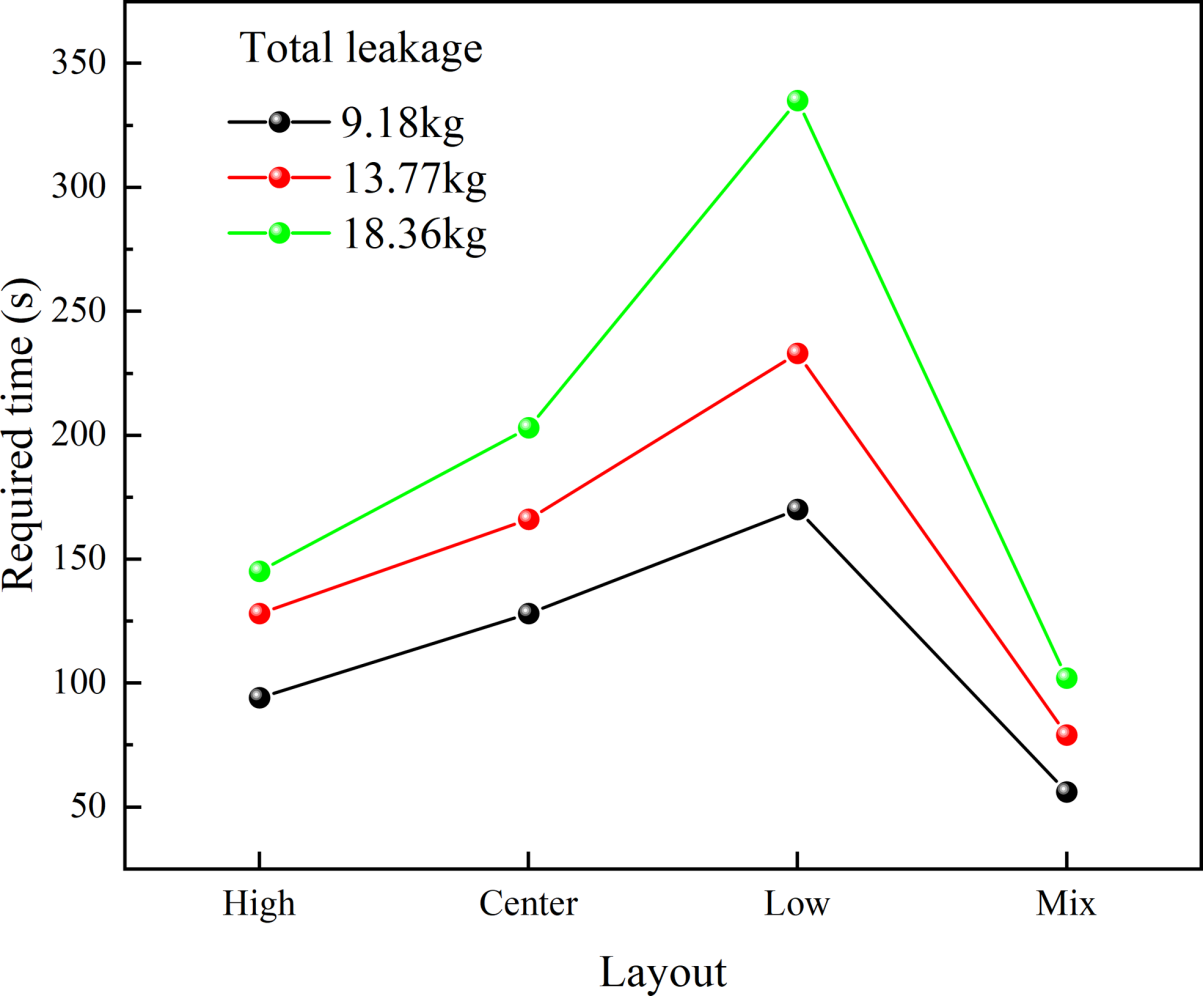
Required time for different total leakage mass.

Figure 20 shows the variation of hazardous gas cloud volume under different total leakage mass conditions. The ranking of different ventilation layouts does not change with the total leakage mass increasing. However, the process of exhausting the hazardous gas cloud is significantly delayed. Figure 21 shows the required time variation under different total leakage mass. It can be found that the required time increases with the total leakage mass increasing. However, there are differences in the time increments under different ventilation conditions. The time increment in the low condition is significantly higher than in the other conditions. Centre condition has the second highest time increment after low condition. It shows the significance of improved ventilation. In the case of a significant increase in total leakage mass, longer ventilation times are not required to have a significant effect.

**Figure 22.**
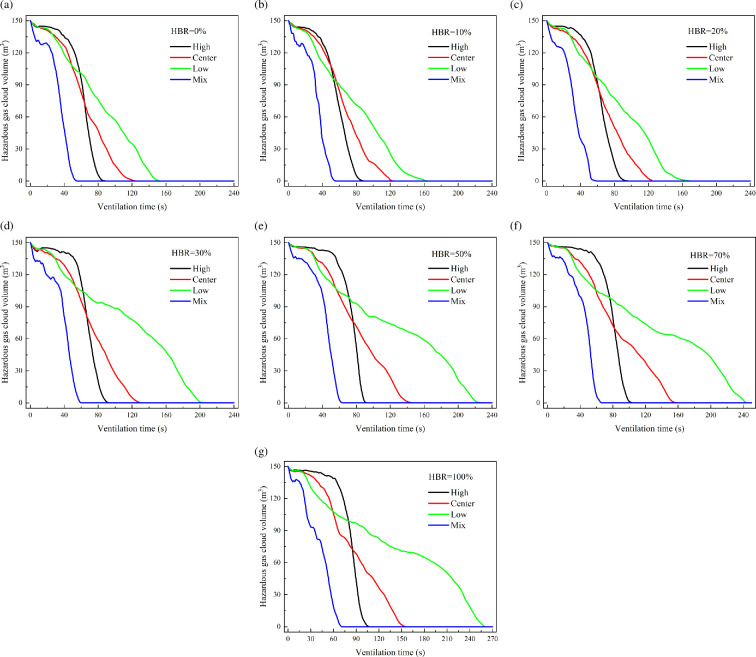
Hazardous gas volume variation under different ventilation conditions.

**Figure 23.**
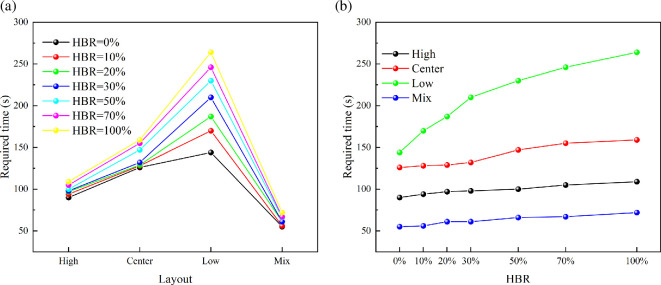
Required time variation under different conditions.

Figure 22 shows the variation of hazardous gas cloud volume under different HBRs. It shows that the exhaust ranking of ventilation layouts also remains the same with different HBRs. The required time to exhaust the gas decreases as shown in figure 23. This pattern can be explained by the inherent properties of leaked gas. Hydrogen has a significantly higher diffusion capacity than methane [25]. Therefore, the diffusion capacity of HBNG increases with HBR increasing. There is no HBNG distribution in ambient environment except for a confined space. For the same leakage time, leaked gas with higher hydrogen proportion diffuses faster driven by the concentration gradient. However, differences in diffusion capacity cause relatively limited time differences. For low, centre, high and mix conditions, the time differences due to HBR changes are 121, 33, 19 and 17 s, respectively. The time difference caused by HBR is more significant only under the low condition. Especially in the current range of engineering applications (HBR < 30%), the time difference is almost negligible. Therefore, the focus should be on analysing the effect of variation in ventilation condition on the exhaust gas process.

#### 3.2.2. Analysis of airflow behaviour under different ventilation conditions

Analysing the physical properties of leaked gas, the density of HBNG is lower than air. The proportion of leaked gas is more in high than in low levels. Therefore, during the ventilation process, the vents are located at a high level to facilitate the exhaust of leaked gas. And for the mix condition, the unsymmetrical vents make the exhaust process more complicated.

**Figure 24.**
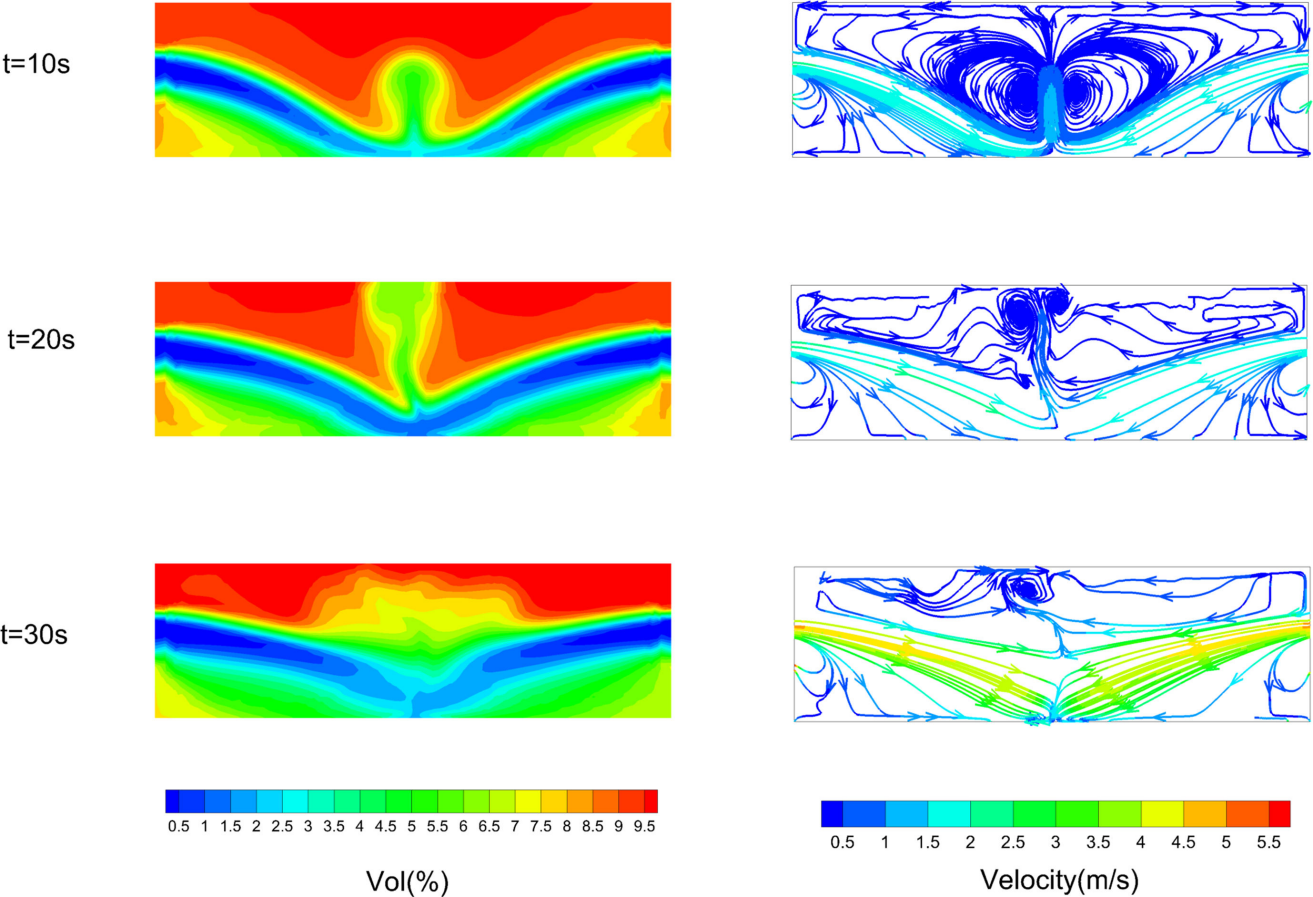
Leaked gas variation process on *Z* = 0 m plane under centre condition.

**Figure 25.**
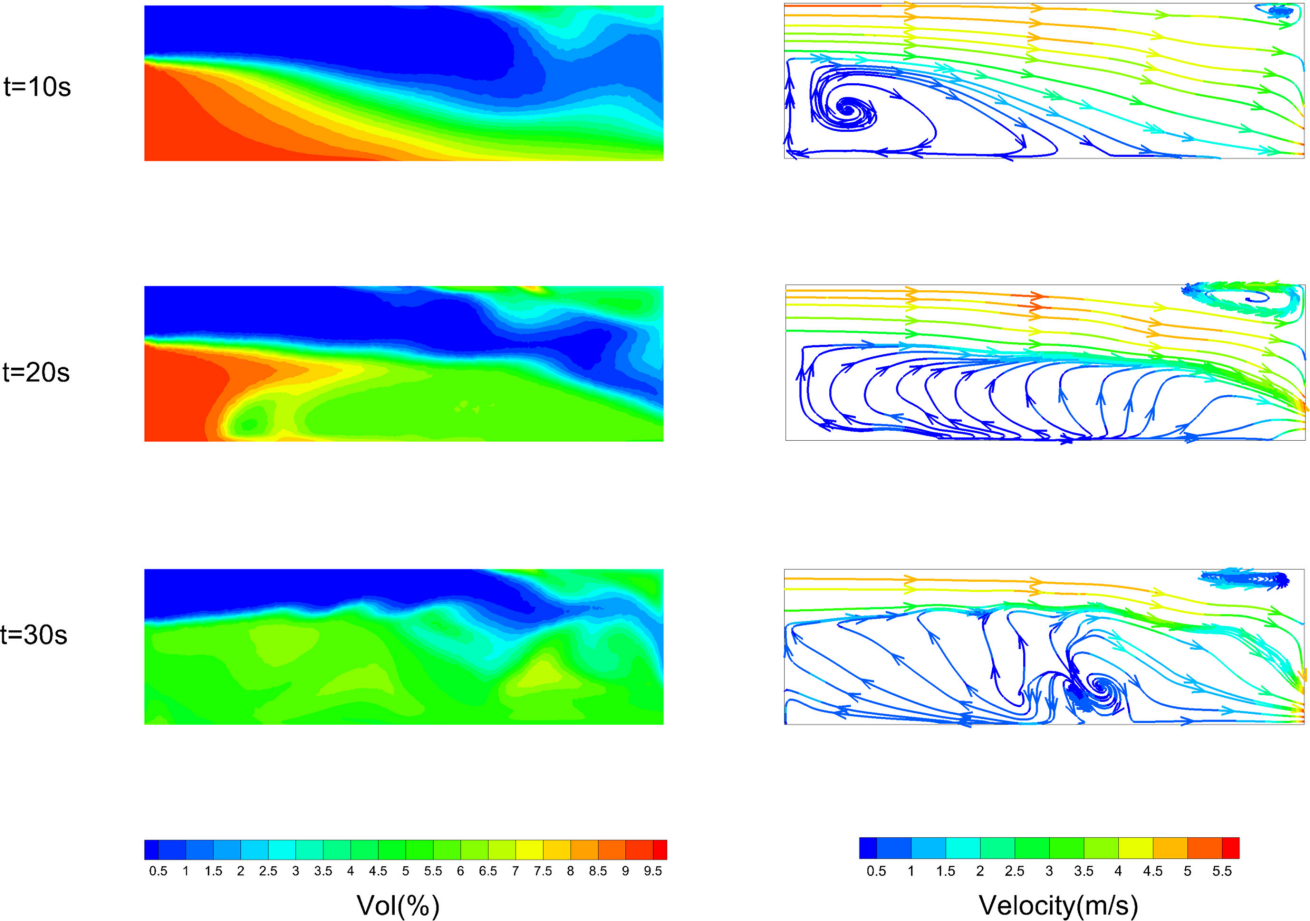
Leaked gas variation process on *Z* = 0 m plane under mix condition.

Figures 24 and 25 show the volume fraction of leaked gas and streamlines in the *Z* = 0 m plane under centre and mix conditions. From figure 24, it can be found that vol(%) decreases in the middle height area at first. The corresponding streamlines point from outside towards inside. It shows that outside air is pouring into the confined space. Indoor leaked gas diffuses towards the outside under the effect of concentration gradient. At this point, the direction of leakage gas diffusion is not consistent with the direction of airflow. It can be inferred that the vents would show inflow of air and outflow of leaked gas.

The flow characteristics under mix and centre conditions are completely different. From figure 25, it can be noticed that vol(%) decreases first in the upper left area near window 1 and then in other areas. In the upper left area, the volume fraction decreases to about 1% within 10 s. After that the area greater than 9% gradually decreases. On the other hand, streamlines move from the upper left (window 1) towards the lower right (window 2). The velocity of gas flow in this path is also greater than in other areas. It can be inferred that window 1 rushes a large amount of air into the interior, which pushes leaked gas from the interior to vent out by window 2. This inference is verified by analysing the flow constitutions of vents following.

**Figure 26.**
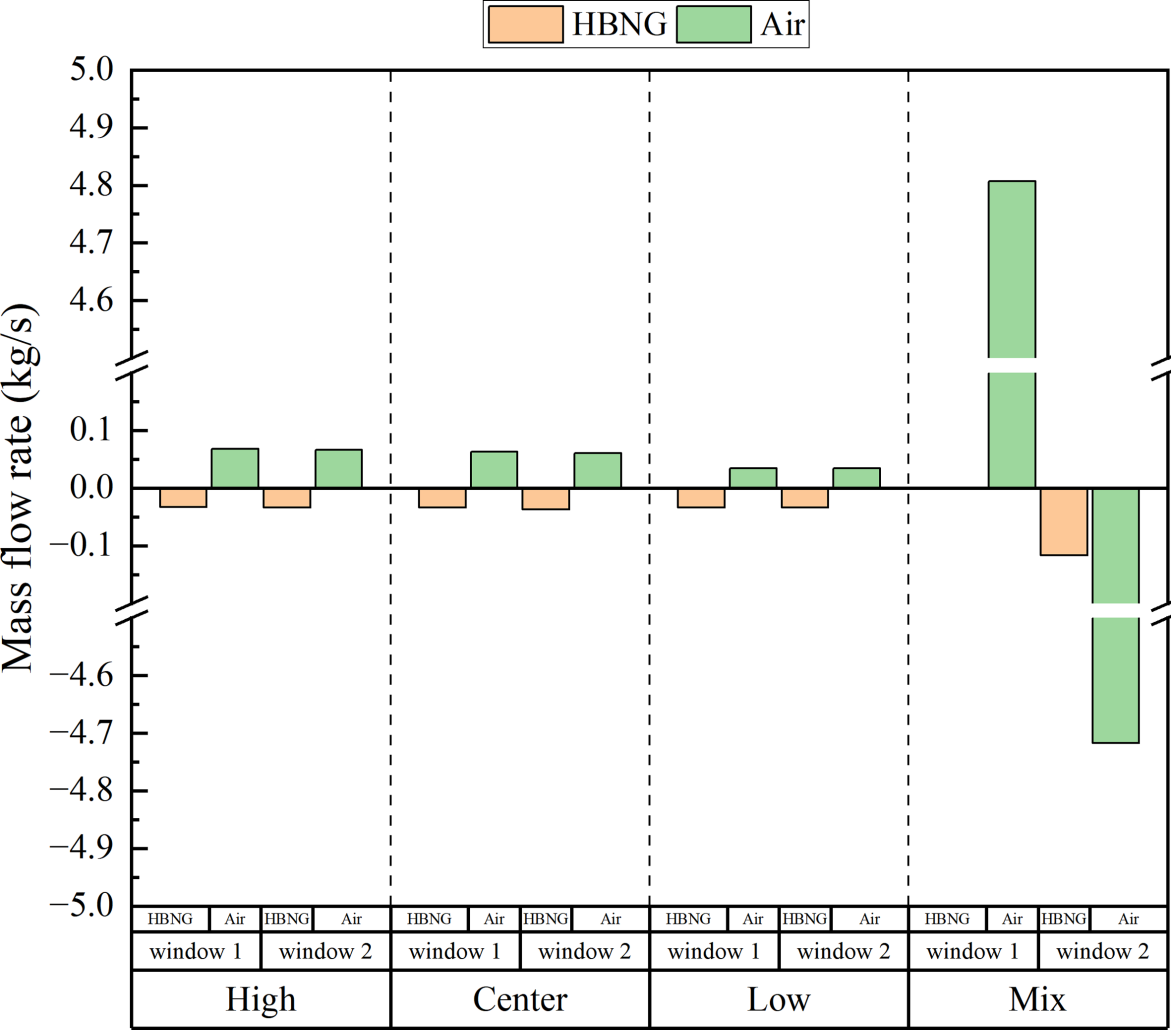
Mass flow constitutions under different ventilation conditions.

Figure 26 shows the constitutions of average flow at all vents for HBR = 0%. Positive values represent inflow from external space and negative values represent outflow from internal space. It can be found that flow constitutions of window 1 and window 2 for high, centre and low conditions are essentially equal. Each vent has air inflow and HBNG outflow. The maximum airflow rate is 0.0627 kg s^−1^, and the maximum HBNG flow rate is 0.0341 kg s^−1^. And for mix conditions, the flow constitutions are completely different. Window 1 shows almost no HBNG outflow, and window 2 shows HBNG outflow at 0.117 kg s^−1^. Air inflow and outflow far exceed that of HBNG, reaching about 4.7 kg s^−1^. This validates previous inferences. The large amount of airflow under mix conditions carries HBNG movement. This causes the hazardous gas cloud to dissipate rapidly.

**Figure 27.**
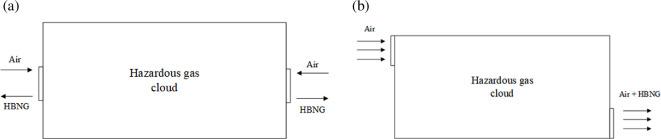
Gas flow behaviour diagram of different ventilation conditions.

The flow behaviour diagram is shown in figure 27. For symmetrical ventilation conditions such as high, centre and low, the vents all flow into air and out of HBNG. In connection with figures 26 and 25, the higher the airflow rate, the better the ventilation. Airflow under the low condition is significantly lower than under other conditions, so hazardous gas cloud dissipation is the slowest. For unsymmetrical ventilation condition, the mix condition, a large amount of air flows from left side and carries leaked HBNG out from right side, creating a behaviour of air displacing leaked HBNG. Airflow rate also far exceeds that of symmetric conditions. As a result, the mix condition causes the best ventilation performance.

**Figure 28.**
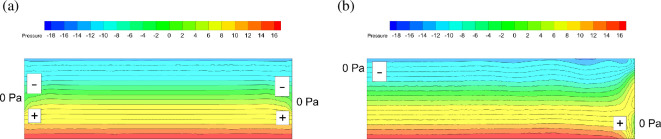
Pressure distribution of different ventilation conditions.

The reason for the airflow pattern can be analysed based on the pressure distribution on *Z* = 0 m. The pressure distribution under centre and mix conditions after ventilating for 10 s is shown in figure 28. It can be found that the upper part is a negative pressure zone and the lower part is a positive pressure zone. For the symmetric centre condition, the outside gauge pressure is 0 Pa. Along the direction of pressure gradient, outside air will pour in from 0 Pa area towards the negative pressure area and then gas flow exchanges momentum in the middle position. Ultimately, the streamline shown in figure 24 is formed. The leaked gas just relies on the concentration gradient to diffuse outward. For unsymmetrical mix conditions, diffusion of leaked gas can be enhanced by the convective effect. The negative pressure zone in the upper left part of the space makes air pour inward from 0 Pa area. The positive pressure zone in the lower right part makes the leakage gas flow towards 0 Pa area. These features lead to the streamline as shown in figure 25. In the lower right part, the concentration gradient of leaked gas also points outside, consistent with the airflow direction. The diffusion of leaked gas can be enhanced. Ultimately, it makes exhausting leaked gas faster.

## 4. Conclusions

In this study, HBNG leakage and ventilation processes under different HBRs and ventilation conditions are simulated by CFD. Based on the analysis of hazardous gas cloud volume evolution, the following main conclusions are obtained.

1. The HBNG leakage process is analysed for different leakage direction, leakage diameter and HBRs. In all conditions, hazardous gas cloud volume is almost 0 m^3^ at first and then rises rapidly at a certain moment until the hazardous gas cloud fills the whole space. With larger leakage diameter, the time required for the hazardous gas cloud reaching 150 m^3^ is shorter. The accumulation rate of the hazardous gas cloud increases with leakage angle. Vertical upward leakage accumulates fastest. The difference in required time under each leakage direction is less than 5%. It is not significant for the whole leakage process. The reason is that the main form of airflow for leakage is plume. The effect of initial momentum is small.
2. The required time for hazardous gas cloud filling the entire space just increases slightly with HBR. HBR and required time show an approximately linear relationship. The time difference caused by HBR = 0 and 100% is about 360 s. Especially for HBR under 30%, the time difference can only reach 15 s. The main reason for the difference is the variation of LEL with HBR. For the rapid increase stage of hazardous gas cloud volume, the distribution of leaked HBNG in the space is relatively uniform except for the near field of leakage location. All parts of space tend to reach LEL at the same moment. It leads to a rapid increase of hazardous gas cloud after a specific moment.
3. The HBNG ventilation process is analysed for different ventilation conditions. The required time for the hazardous gas cloud to reach 0 m^3^ increases with the total leakage mass. The required time for this process decreases with increasing HBR. The main reason is the increasing of diffusion capacity with HBR. For different ventilation conditions, the order of required time for this process is: low > centre > high > mix. The mix condition takes only about 30% required time under low condition. The differences are mainly in the behaviour of airflow. When vents are symmetrical, the ventilation efficiency rises with vent height. Whereas ventilation efficiency under symmetrical conditions is significantly lower than under unsymmetrical condition. When vents are unsymmetrical as in the mix condition, a large amount of air flows into the space to displace leaked HBNG. Convection promotes the diffusion of leaked gas. This flow behaviour significantly improves the ventilation efficiency.

The above conclusions have some reference value for the safe utilization of HBNG. Without considering the ignition probability, HBR has little influence on hazardous gas cloud evolution and does not need to be considered as a priority. In specific scenarios, one should try to set up mix ventilation conditions to improve the vent efficiency of hazardous gas cloud.

However, there are some limitations in this study. As a matter of fact, the situation of thermal boundaries also has some influence on gas behaviour, while the boundaries in this study are considered according to constant temperature conditions. Subsequent studies may consider the effect of this condition variation and verify it with experiments.

## Data Availability

The data that support the findings of this study are available on Dryad [26].
